# Genome-Wide Analysis of Histidine Repeats Reveals Their Role in the Localization of Human Proteins to the Nuclear Speckles Compartment

**DOI:** 10.1371/journal.pgen.1000397

**Published:** 2009-03-06

**Authors:** Eulàlia Salichs, Alice Ledda, Loris Mularoni, M. Mar Albà, Susana de la Luna

**Affiliations:** 1 Genes and Disease Program, Centre de Regulació Genòmica (CRG), Barcelona, Spain; 2 El Centro de Investigación Biomédica en Red de Enfermedades Raras (CIBERER), Barcelona, Spain; 3 Biomedical Informatics Research Program, Institut Municipal d'Investigació Mèdica-IMIM, Barcelona, Spain; 4 Universitat Pompeu Fabra, Barcelona, Spain; 5 Institució Catalana de Recerca i Estudis Avançats (ICREA), Barcelona, Spain; Johns Hopkins University School of Medicine, United States of America

## Abstract

Single amino acid repeats are prevalent in eukaryote organisms, although the role of many such sequences is still poorly understood. We have performed a comprehensive analysis of the proteins containing homopolymeric histidine tracts in the human genome and identified 86 human proteins that contain stretches of five or more histidines. Most of them are endowed with DNA- and RNA-related functions, and, in addition, there is an overrepresentation of proteins expressed in the brain and/or nervous system development. An analysis of their subcellular localization shows that 15 of the 22 nuclear proteins identified accumulate in the nuclear subcompartment known as nuclear speckles. This localization is lost when the histidine repeat is deleted, and significantly, closely related paralogous proteins without histidine repeats also fail to localize to nuclear speckles. Hence, the histidine tract appears to be directly involved in targeting proteins to this compartment. The removal of DNA-binding domains or treatment with RNA polymerase II inhibitors induces the re-localization of several polyhistidine-containing proteins from the nucleoplasm to nuclear speckles. These findings highlight the dynamic relationship between sites of transcription and nuclear speckles. Therefore, we define the histidine repeats as a novel targeting signal for nuclear speckles, and we suggest that these repeats are a way of generating evolutionary diversification in gene duplicates. These data contribute to our better understanding of the physiological role of single amino acid repeats in proteins.

## Author Summary

Single amino acid repeats are common in eukaryotic proteins. Some of them are associated with developmental and neurodegenerative disorders in humans, suggesting that they play important functions. However, the role of many of these repeats is unknown. Here, we have studied histidine repeats from a bioinformatics as well as a functional point of view. We found that only 86 proteins in the human genome contain stretches of five or more histidines, and that most of these proteins have functions related with RNA synthesis. When studying where these proteins localize in the cell, we found that a significant proportion accumulate in a subnuclear organelle known as nuclear speckles, via the histidine repeat. This is a structure where proteins related to the synthesis and processing of RNA accumulate. In some cases, the localization is transient and depends on the transcriptional requirements of the cell. Our findings are important because they identify a common cellular function for stretches of histidine residues, and they support the notion that histidine repeats contribute to generate evolutionary diversification. Finally, and considering that some of the proteins with histidine stretches are key elements in essential developmental processes, variation in these repeats would be expected to contribute to human disease.

## Introduction

Single amino acid repeats (SARs), also known as homopolymeric tracts, are very common in eukaryotes [1] and between 18–20% of proteins in the human genome contain such repetitive sequences [2]. Although most of them are thought to be functionally neutral, recent evidence suggests they may play important functional or structural roles. Indeed, there is an overrepresentation of SARs-containing proteins (SARPs) among transcription factors, kinases and proteins required for development [2]–[5]. The intrinsic disorder of such repeats converts them into flexible spacer elements between individual folded domains, allowing SARPs to associate in large, multiprotein complexes [5],[6]. In addition, it is thought that disordered regions can bind to multiple targets with weak affinity, an ideal property for elements involved in transcriptional and signal transduction processes [7].

Homopolymeric tracts are often encoded by trinucleotide repeats, a class of microsatellites. Their repetitive nature facilitates DNA replication slippage, and the expansion or contraction of the repeats (for review, see [8]). Although genetic variability of these repeats provides a substrate for adaptive evolution [9],[10], uncontrolled expansion of such unstable regions within coding sequences has been associated with a number of developmental and inherited neurodegenerative disorders [2],[11], as well as with several types of cancer [12]. For example, polyglutamine expansions have been associated with Huntington's disease and certain types of spinocerebellar ataxia (for review, see [11]). In addition, alanine repeats are related to several developmental disorders (for review, see [13]), and aspartate hyperexpansions with two types of dysplasia and osteoarthritis [14],[15]. Some of the mechanisms thought to underlie the pathogenic effects of expanded tracts involve the deregulation of transcriptional activity and the formation of toxic protein aggregates (for review, see [11],[16]). Nevertheless, the functions of many homopeptidic segments found in proteins have not yet been elucidated.

Among homopolymeric tracts, histidine (His) repeats are relatively rare [5]. However, their frequency increases from about 1.4% to 4.3% when we consider repeats of at least 8 instead of 5 residues, indicating that they are generally longer than other types of SARs [4]. The physicochemical properties of His make it a versatile amino acid that can fulfill different roles, influencing protein conformation and enzymatic activity. For instance, His-repeats are found in Zn-finger domains that are implicated in interactions between nucleic acids and proteins (for review, see [17]), and a His-stretch has been described as a protein interacting surface of the transcriptional regulator cyclin T1 [18],[19]. Nevertheless, there is still no clear function associated to His homopeptides. We previously described the His-repeat in the DYRK1A protein kinase as both necessary and sufficient to target this protein to nuclear speckles [20]. A protein segment containing a His-tract is also involved in the accumulation of cyclin T1 in these nuclear structures [20],[21]. These results provided the first evidence that His-repeats may act as nuclear speckle-targeting signals, although the extent to which this was true in other proteins remained to be determined.

Nuclear speckles (also known as the splicing factor compartment -SFC- or as interchromatin granule clusters -IGCs-) are subnuclear structures defined as compartments in which components of the RNA splicing machinery are stored and assembled (for review, see [22]). They mainly contain splicing factors (snRNPs and serine/arginine-rich (SR) proteins), as well as transcription factors, 3′-RNA processing factors, translation factors, ribosomal proteins, a subpopulation of the RNA polymerase II and some kinases and phosphatases [23],[24]. Like other nuclear bodies, nuclear speckles are highly dynamic structures that change in number, shape and size depending on the transcriptional state and the phase of the cell cycle [22].

Here, we have performed an in-depth analysis of polyHis-containing proteins in the human genome. A significant fraction of the proteins identified are transcription factors and developmental proteins with a nuclear phase. The subcellular localization of several of these proteins shows that most of them accumulate in nuclear speckles through their His-repeat. The presence of DNA-binding or protein-protein interaction domains, and the transcriptional state of the cell, are factors that affect the retention of transcription factors with His-repeats in nuclear speckles, illustrating the dynamic behavior of these proteins. Together, these results define the His-repeat as a novel and general targeting signal for nuclear speckles.

## Results

### A Repeat of 6 His Residues Is Sufficient to Direct a Heterologous Protein to the Nuclear Speckles

**Figure 1:**
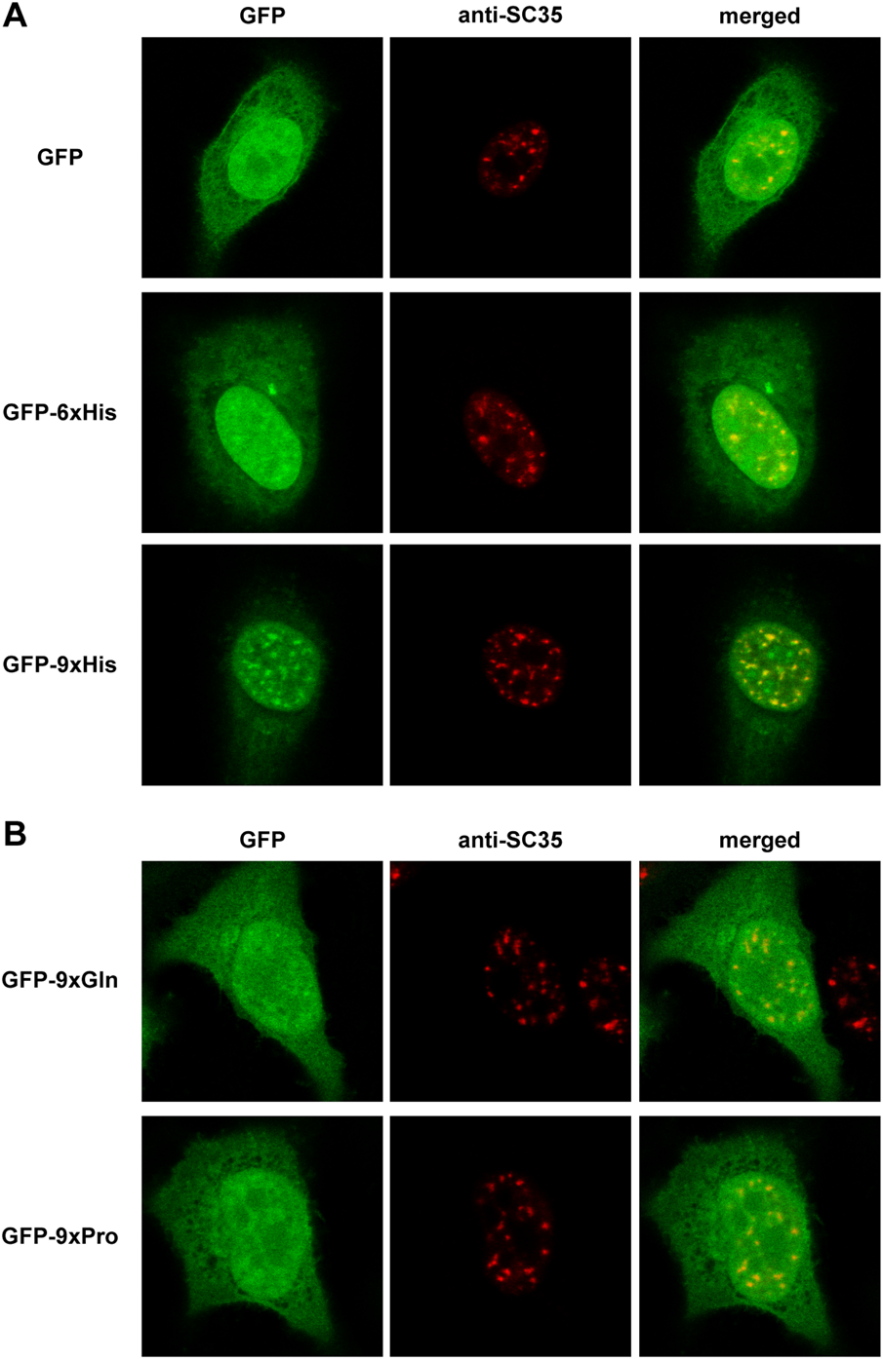
The ability of a His-repeat to direct a heterologous protein to the nuclear speckles depends on the number of His residues in the tract. A) HeLa cells were transfected with expression plasmids encoding fusion proteins of GFP with 6 or 9 His residues. B) Cells were transfected with expression plasmids encoding fusion proteins of GFP with 9 Pro or Gln residues, as indicated. At 48 h post-transfection, the localization of the fusion proteins was analyzed by direct fluorescence (left column, green) and by indirect immunofluorescence for SC35 (middle column, red). The merged images are also shown (left column), and the unfused GFP protein was used as a control. In all cases, co-localization with the endogenous marker was determined by confocal imaging.

For a typical protein of 400 amino acids and of average composition, a run of any individual amino acid is significant if there are 5 or more consecutive residues [25]. Following this premise, we established a threshold of 5 His residues to determine the minimum number of His necessary for a His-containing protein to accumulate in nuclear speckles. We generated plasmids to express green fluorescent protein (GFP) fusion proteins with 5, 6, 7, 8 or 9 His, and we analyzed the subcellular localization of these fusion proteins by direct fluorescence in transfected HeLa cells. Nuclear speckles were identified by indirect immunofluorescence with an antibody against the splicing factor SC35, an endogenous marker of the nuclear speckles compartment [26]. No significant differences in the staining pattern were observed when GFP and GFP-5xHis were compared (Figure S1). However, from the 6xHis constructs onwards, a positive relationship was detected between the accumulation in nuclear speckles and the length of the His-tract. While GFP-6xHis only weakly concentrated in SC35-positive speckles, this association became stronger as the number of His residues increased, and it was clearly evident with a fusion protein containing 9 His (Figure 1A and S1).

To confirm that the GFP-His fusions almost completely co-localized with SC35 positive speckles, we carried out an immunofluorescence analysis with protein markers of other subnuclear compartments that are compatible with such staining, including promyelocytic leukemia (PML) bodies (for review, see [27]), Sumo-bodies (for review, see [28]) or paraspeckles [29]. No co-localization between the GFP-9xHis fusion protein and any of the protein markers (PML, Sumo1, PSP1) was detected (Figure S2).

Finally, the subnuclear localization of GFP fusion proteins with polyproline or polyglutamine tracts, which are particularly enriched in transcription factors [4] and that have been shown to be functional as transcriptional activators [30], was also analyzed. These fusion proteins showed nucleoplasmic staining and no colocalization with SC35 (Figure 1B), in agreement with previous results with longer amino acid tracts [31]. Therefore, His homopolymeric tracts seem to specifically accumulate in the nuclear speckles compartment.

### The Distribution of His-Repeats in the Human Proteome

To extrapolate these results to real proteins, we performed a bioinformatics screen of the Ensembl database [32] to identify all the human proteins containing at least one His-repeat of 5 or more residues. The lower-limit of 5 His residues was set to cover all possible functionally significant repeats [25]. Our search identified 86 Ensembl genes (Table S1). As some of the proteins encoded by these genes contained more than one repeat, there was a total of 99 repeats with 5 residues or more. The average size of the His-repeats was 7.5, with the longest repeat containing 15 residues (LOC730417). The majority of the repeats were well conserved in the corresponding mouse orthologous proteins; 54% showed exactly the same length and 30% differed in only one or two repeat units. When more than one His-repeat was present in a protein, they were generally very close to each other such that they could be considered as “extended” His-repeat tracts (for instance, H_4_GNSSH_13_ in DYRK1A). Thus, we defined “extended” tracts as regions that contained at least one pure His-repeat of 5 residues or more, that had His residues at the start and/or end of the tract, and that contained other “interrupting” residues (often P, Q, G, S, A) which covered <50% of the tract. Such extended tracts were present in half of the proteins containing pure His-repeats (43 out of 86). Significantly, none of the His-repeats were situated within characterized protein domains and unlike other repeats [4], we did not find them preferentially located at the amino-, carboxy-, or central part of the proteins.

**Figure 2:**
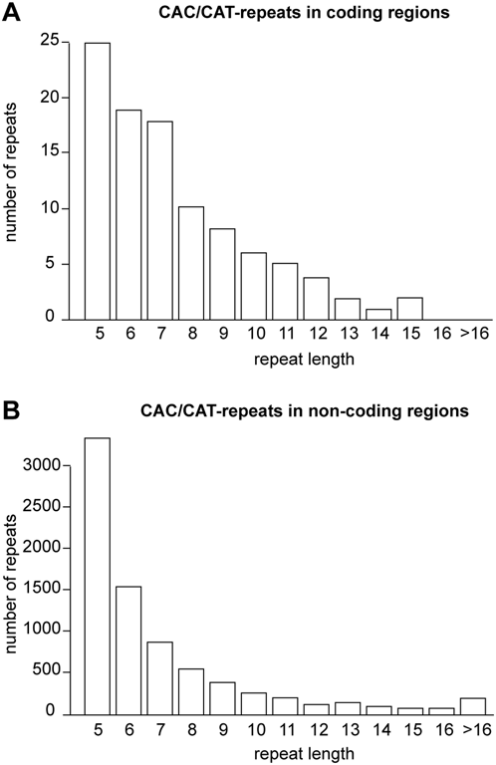
Distribution of CAC/CAT repeat sizes in coding (A) and non-coding (B) regions.

We compared the length distribution of His-repeats in coding sequences to that of equivalent sequences in non-coding regions, the latter defined as sequences containing at least five tandem CAY (CAC or CAT: His encoding triplets). Accordingly, we identified 7815 such repeats in non-coding genomic regions. Interestingly, although much longer repeats existed in the non-coding regions (the longest was 154 trinucleotides), their average size (7.24) was smaller than in coding regions. Indeed, the distribution of the repeat size was significantly different between coding and non-coding sequences (*p*-value = 0.003, non-parametric Kolmogorov-Smirnov test). In coding sequences, there was an under-representation of short repeats (size 5) with respect to longer repeats (around 7) when compared to non-coding sequences (Figure 2A and 2B, respectively). As the length distribution of non-coding repeats is likely to reflect neutral mutational processes, this difference points to selective retention of relatively long His-repeats in protein sequences.

### His-Repeats Are Overrepresented in Nuclear Proteins

**Figure 3:**
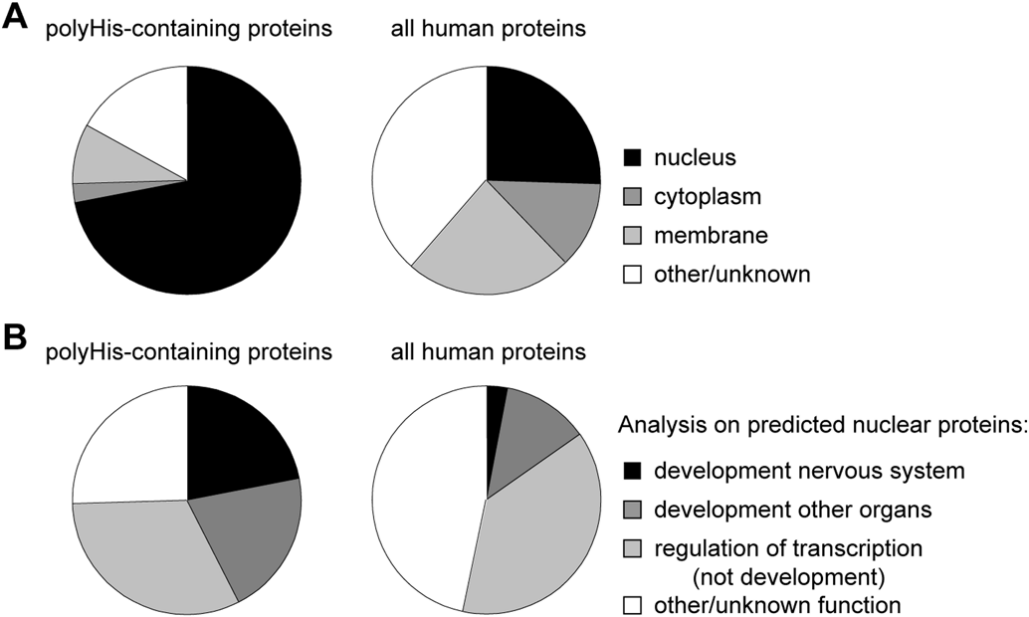
Gene Ontology distribution of polyHis-containing proteins. A) Distribution of genes annotated as ‘nucleus’, ‘cytoplasm’ (excluding ‘nucleus’) and ‘membrane (excluding ‘nucleus’ and ‘cytoplasm’). B) Distribution of the main functional groups in nuclear His-repeat containing proteins and a comparison with the same groups in the complete gene dataset (see Materials and Methods for more details).

The population of proteins containing other types of amino acid repeats, such as polyglutamine, polyalanine, polyglycine, polyserine and polyproline, is enriched in transcription factors [4]. We examined whether any such bias in Gene Ontology terms (GO; [33]) existed in the gene dataset encoding His-repeats. Among proteins containing His-repeats there was a strong over-representation of nuclear proteins (72% with respect to 26% in the complete protein dataset, *p*-value<10^−5^, Figure 3A). In addition, 75% of the His repeat-containing nuclear proteins were also annotated with the GO term ‘regulation of transcription’, in comparison with 49% of those in the complete nuclear protein dataset. Even more striking was the strong over-representation of developmental factors among nuclear proteins with His-repeats, especially those involved in the development of the nervous system (22% with respect to 3% in the complete gene dataset, *p*-value<10^−5^, Figure 3B). This finding is in agreement with previous work [34] and it might be linked to the fact that increased formation of homopolymeric runs in human proteins may be a recent evolutionary event, concomitant with complex brain development [2].

### The His-Repeat Is a Novel Nuclear Speckle-Directing Sequence

**Figure 4:**
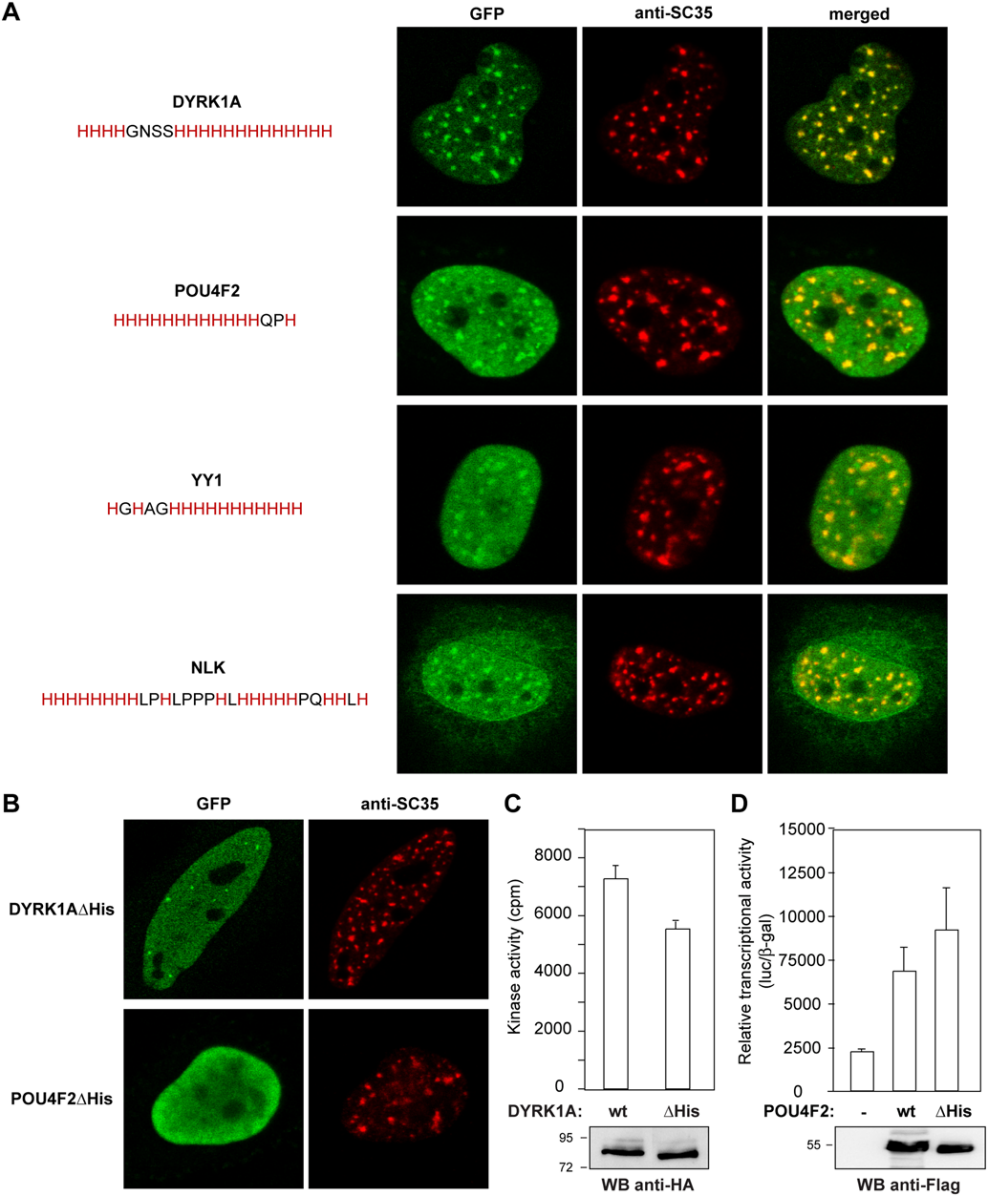
The His-repeat is a novel nuclear speckle targeting signal. A) HeLa cells were transfected with the expression plasmids for the fusion proteins GFP-DYRK1A, GFP-POU4F2, GFP-YY1 and GFP-NLK. Cells were immunostained for SC35 to visualize the nuclear speckles (middle column, red) and GFP fusion proteins were visualized directly by fluorescence microscopy (left column, green). Merged images are shown (right column). B) HeLa cells were transfected with the expression plasmids for HA-DYRK1AΔHis and Flag-POU4F2ΔHis, and the cells were immunostained for DYRK1A or POU4F2 (left column) and for SC35 to detect nuclear speckles (middle column). C) Soluble extracts from cells expressing HA-DYRK1A or HA-DYRK1AΔHis were subjected to immunoprecipitation with anti-HA and then *in vitro* kinase activity on the DYRKtide peptide was assayed. Samples were analyzed in Western blots probed with anti-HA. D) Cells were co-transfected with pGL2-3xBrn3a and pCMV-βgal together with pFlag-POU4F2 wild type (wt) or pFlag-POU4F2ΔHis (ΔHis). Transcriptional activity is presented as the ratio of luciferase and β-galactosidase; values are the means±S.D. of triplicate determinations for each condition in one representative experiment of three performed. The panel shows a Western blot of transfected extracts probed with an anti-Flag antibody.

**Figure 5:**
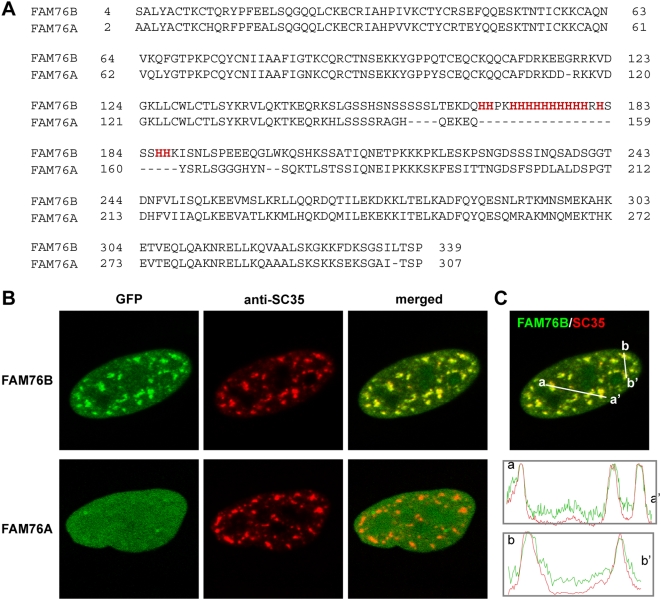
The presence of a His-repeat dictates the different subcellular localization of paralogous proteins. A) Alignment of the primary sequences of the paralogues, FAM76B (NP_653265; hypothetical protein LOC143684) and FAM76A (NP_689873; hypothetical protein LOC199870), obtained with the multiple sequence alignment program “Blast 2 Sequences” (http://www.ncbi.nlm.nih.gov/blast/bl2seq/wblast2.cgi). His residues in FAM76B are highlighted in red. B) HeLa cells were transfected with an expression plasmid encoding FAM76B (upper panel) or FAM76A (lower panel) fused to GFP at their N-terminal. The subcellular localization of the fusion proteins was analyzed by direct fluorescence and their accumulation in nuclear speckles was followed by immunostaining for SC35. C) Using the lines on the merged image, fluorescence intensity profiles were obtained for GFP (green) and SC35 (red).

The GO terms analysis indicated that most of the polyHis-containing proteins are nuclear proteins, and therefore they might be targeted to nuclear speckles. Thus, we analyzed the distribution of a group of the nuclear-annotated proteins with pure His-repeats of different lengths (longer than 5 residues) and several proteins with extended repeats. The subcellular localization of the His-containing proteins was analyzed by generating GFP fusion proteins with the open reading frames of candidate proteins in a mammalian expression vector. The subcellular distribution of the fusion proteins was analyzed by direct fluorescence in transient transfected cells and nuclear speckles were identified by anti-SC35 staining. As previously described for cyclin T1 and DYRK1A [20], other polyHis-containing proteins also showed punctate nuclear staining that co-localized with SC35, such as the transcription factors POU4F2 or YY1, or the protein kinase NLK (Figure 4A). Fluorescence images revealed differences in the staining patterns for the His-repeats-containing proteins, with some of them showing more nucleoplasmic staining than others (Figure 4A; see other examples in Figures 5–8). The His-repeat seemed to be necessary for this subnuclear localization since deletion of the polyHis segment alone from POU4F2 or DYRK1A (the extended His-repeat) completely abrogated the accumulation of these proteins in SC35-labelled nuclear speckles (Figure 4B). These results indicate that the His-repeat can act as a nuclear speckle localization signal.

**Figure 6 pgen-1000397-g006:**
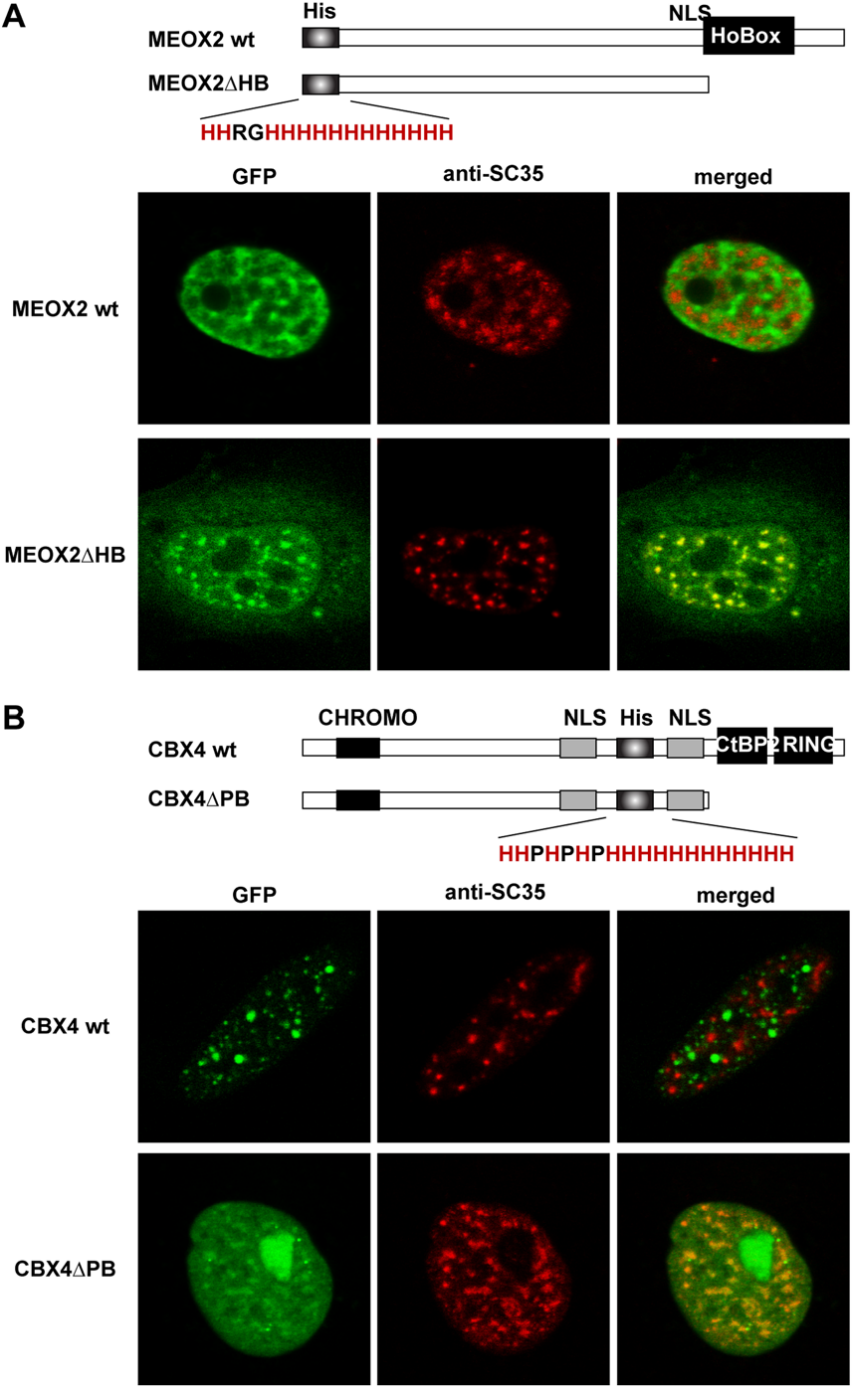
The accumulation in nuclear speckles of some polyHis-containing proteins depends on the presence of other interacting domains. A) HeLa cells were transfected with the expression plasmids for wild type GFP-MEOX2 or the mutant GFP-MEOX2ΔHB as indicated (see scheme; His: His-repeat; NLS: nuclear localization signal; HoBox: homeobox domain). B) HeLa cells were transfected with the expression plasmids for GFP-CBX4 wild type or GFP-CBX4ΔPB as indicated (see scheme: CHROMO, chromatin organization modifier domain; His, His-repeat; NLS, nuclear localization signal; CtBP2, CtBP binding domain; and RING1, RING1-interacting domain). In A) and B), the subcellular localization of the GFP-fusion proteins was analyzed by direct fluorescence (left column, green) and their accumulation in nuclear speckles by immunofluorescence for SC35 (middle column, red).

**Figure 7 pgen-1000397-g007:**
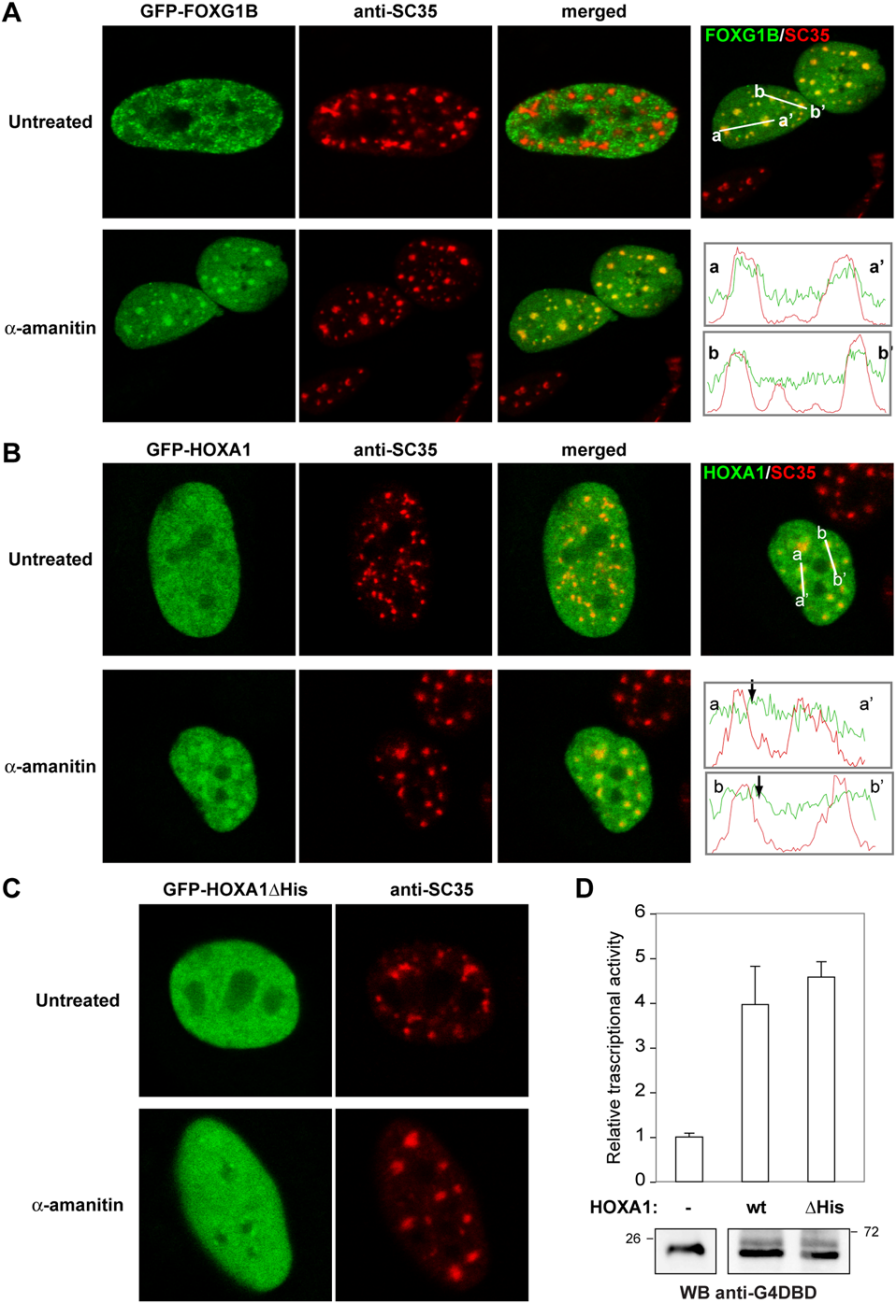
The transcriptional state of the cell determines whether some polyHis transcription factors accumulate in nuclear speckles. A, B) HeLa cells were transfected with the expression plasmids encoding the GFP-FOXG1B (A) and GFP-HOXA1 (B) fusion proteins. At 36 h post-transfection, cells were treated with α-amanitin for 5 h to inhibit transcription and then processed for SC35 immunofluorescence. Fluorescence intensity profiles are shown for GFP (green) and SC35 (red), obtained from the lines on the merged images. C) The panels show the results for the same type of experiment performed on mutant HOXA1ΔHis in which the His-tract has been eliminated (see scheme: His, His-repeat; NLS, nuclear localization signal; HoBox, homeobox). D) Cells were co-transfected with pE1bG4-luc and pCMV-RNL together with pG4-DBD (-), pG4-HOXA1 wild type (wt) or pG4-HOXA1ΔHis (ΔHis), and luciferase activity was measured in triplicate plates. Values were corrected for transfection efficiency as measured by Renilla activity. Data is presented as the induction of luciferase activity above the G4-DBD transfection and the values are the means±S.D. of triplicate determinations for each condition in a representative experiment of a minimum of two performed. The panel shows a Western blot analysis of transfected extracts with an anti-Gal4-DBD antibody.

**Figure 8:**
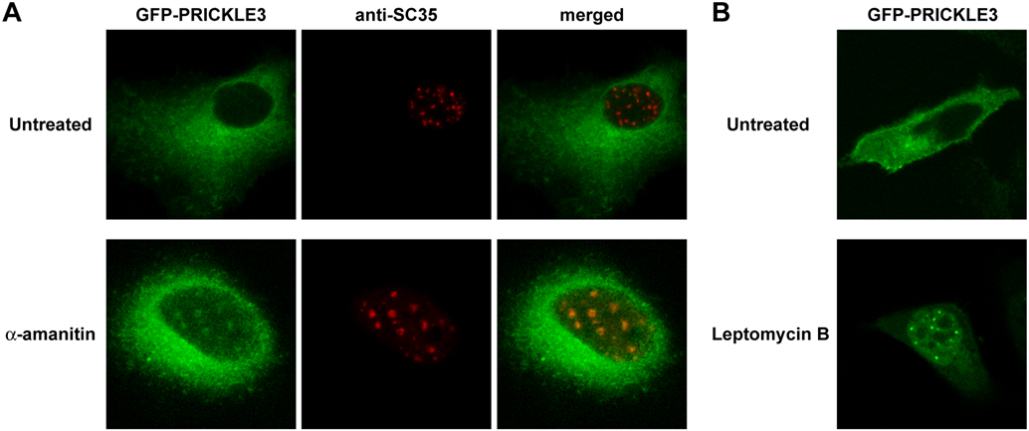
The His-tract participates in the dynamic properties of polyHis-containing proteins. A) HeLa cells were transfected with the expression plasmids encoding GFP-PRICKLE3. Cells were treated with α-amanitin for 5 h to inhibit transcription and then processed for SC35 immunofluorescence. B) HeLa cells expressing the GFP-PRICKLE3 fusion protein were mock-treated or exposed to leptomycin B for 5 h, 24 h after transfection. The subcellular localization of the fusion protein was analyzed by direct fluorescence. Note that PRICKLE3 is detected in the cytosol in untreated cells but it accumulates in the nucleus, nucleoplasm and nuclear speckles in response to the inhibitor of nuclear export.

Moreover, deletion of the His-repeat did not interfere with the biochemical function of the protein, that is “kinase” for DYRK1A or “transcriptional activator” for POU4F2 (Figure 4C and 4D, respectively). Similar results were obtained when the His-repeat was deleted in NLK (Figure S3). These data indicate that the deletion has not induced a general alteration of protein structure, and further suggest that the His-tract conveys a novel behavior to the host protein without affecting its basic activity.

### His-Repeats and Gene Duplication

Interestingly, a significant fraction (64%) of the genes encoding proteins with His-repeats had closely-related paralogues in the human genome. According to Ensembl annotations, 74% of them had been presumably formed by gene duplication at the dawn of vertebrate evolution (Table S2). However, in most cases none of the paralogues contained a similar His-repeat in their primary sequence. This indicates that the repeat had only later appeared in one of the duplicate copies, probably by duplication slippage. To approximately date their appearance, we inspected all the orthologous and paralogous vertebrate proteins in Ensembl for the presence of similar His-repeats. In 11 out of 39 cases, the repeat was found in all vertebrate orthologues but in none of the paralogues, indicating that they arose soon after the duplication event. However, the dominant class was repeats formed at the base of the placental mammals (14 cases). Notably, a large number of alanine and glycine repeats are also proposed to be specific to mammals [35]–[37]. Indeed, the increased repeat expansion in this clade may be related to the increased GC content [38],[39]. Finally, the His-repeats in the *BMP2K* and *PBXIP1* genes were restricted to primates, suggesting they arose relatively recently.

### Paralogous Proteins without His-Repeats Fail to Localize in Nuclear Speckles

Given the significant number of polyHis-containing proteins with paralogous proteins without His-repeats, we reasoned that if the His-repeat were responsible for their accumulation in speckles then the paralogous copy without the repeat should not be found in this subnuclear compartment. To confirm this hypothesis, we examined the FAM76A and FAM76B pair of paralogues. A sequence alignment of these two proteins highlighted their high degree of conservation, except in the region containing the His-repeat (Figure 5A). As hypothesized, the paralogue without the His-tract, FAM76A, presented a diffuse nucleoplasmic staining, while the protein with the polyHis segment, FAM76B, accumulated in nuclear speckles (Figure 5B). Similar results were obtained for other pairs of paralogous proteins such as DYRK1A/DYRK1B or POU4F2/POU4F3 (Figure S4). Thus, these findings further indicate that the His-repeats in these proteins are necessary for their localization to nuclear speckles.

### The Subcellular Localization of PolyHis-Containing Proteins Depends on other Domains Present in the Proteins

The initial analysis of the nuclear localization of polyHis-containing candidates revealed that some proteins did not apparently localize to nuclear speckles. These proteins contained other protein domains such as DNA binding domains or protein-protein interacting regions. For instance, the transcription factors MEOX2 and OTX1 harbor a homeobox DNA-binding domain in their C- and N-terminal regions, respectively (Figure 6A and Figure S5). In the case of the Sumo E3 ligase CBX4, its C-terminal region includes domains that interact with the polycomb protein CtBP2 and the transcriptional repressor RING1 (Figure 6B). These domains mediate the localization of CBX4 to subnuclear foci, that are compatible with polycomb bodies [40]. Therefore, we hypothesized that the accumulation of proteins to nuclear speckles may be influenced by other interactions. To confirm this hypothesis, we deleted the DNA binding domain in MEOX2 and assessed its nuclear distribution. Accordingly, while the wild type protein presented the dispersed distribution typical of most transcription factors (Figure 6A), compatible with active transcription sites [41], the mutant protein in which the homeobox was eliminated (MEOX2ΔHB) fully co-localized with SC35 (Figure 6A). Similar results were obtained with the OTX1 transcription factor (Figure S5). In the case of CBX4, we assessed whether deleting the C-terminal fragment spanning the CtBP2 and RING1-interacting domains (CBX4ΔPB) similarly affected its distribution. While the wild type CBX4 protein was present in nuclear foci that were not positive for SC35, the mutant CBX4ΔPB co-localized with SC35 in the nucleus (Figure 6B). These results confirmed that the accumulation of some of the polyHis-containing proteins in nuclear speckles was influenced by their binding to other nuclear components, such as DNA or diverse subnuclear structures. Moreover, they suggest that competition between distinct protein regions dictates the steady state subnuclear localization of the protein.

### Transit through Nuclear Speckles Is a Dynamic Property of PolyHis-Containing Proteins

In mammalian cells, the structure and function of nuclear speckles is sensitive to the transcriptional state of the cell (for review, see [22]). When cells are treated with RNA polymerase II transcription inhibitors, there is a decrease in the splicing activity and a redistribution of the components of speckles, which are recruited to larger and rounder nuclear speckles [42]. Most of the His-containing proteins were transcription factors and since our results showed that DNA binding activity influenced their accumulation in speckles, we wondered whether their failure to localize to this subnuclear compartment might be reverted by inhibiting RNA polymerase II activity. Two proteins, FOXG1B and HOXA1, that did not produce speckled staining at the steady state, co-localized with SC35 in fewer but larger speckles after α-amanitin treatment (Figure 7A and 7B). Interestingly, the diffuse nucleoplasmic distribution of several other transcription factors became punctate in cells treated with α-amanitin, and it overlapped with SC35 staining (Table 1 and Figure S6A). These dynamic changes in distribution could be observed by *in vivo* imaging (Videos S1 and S2). For HOXA1, we noticed that the staining not only overlapped with SC35 foci but it also adopted a “capped structure”, as described for the recently reported S1-1 nuclear domains [43]. We therefore analyzed co-localization with an anti-S1-1 antibody as a marker of this nuclear domain, and we found that the HOXA1 signal co-localized with both the SC35 and the S1-1 staining (Figure S6B). Since nuclear speckles and S1-1 domains have been suggested to be functionally connected [43], it is possible that HOXA1 could traffic between these two subnuclear domains.

**Table 1:** Summary of the results obtained in the analysis of the subcellular localization of polyHis-containing proteins.

Name	His tract	Protein domains	Function
**Cyclin T1**	513-HPSNHHHHHNHHSHKHSH-530	cdk binding domain	Transcription regulator
**POU4F2**	172-HHHHHHHHHHHHQPH-186	POU domain (254–328)	Transcription factor
		Homeobox (346–405)	Differentiation and survival of retinal ganglion cells
**YY1**	65-HGHAGHHHHHHHHHHH-80	Zinc finger (296–320; 325–347;353–377;383–407)	Transcription factor
			Regulation of development and differentiation
**DYRK1A**	590-HHHHGNSSHHHHHHHHHHHHH-610	Kinase domain (159–479)	Ser/Thr protein kinase
			Regulator of cell proliferation and differentiation
**NLK**	14-HHHHHHHHLPHLPPPHLHHHHHPQHHLH-42	Kinase domain (126–415)	Ser/Thr protein kinase
			Regulator of Wnt-signaling pathways
**FAM76B**	167-HHPKHHHHHHHHHHRHSSSHH-187	Not found	Unknown
GSH2	124-HAH HHHHPPQHHHHHH-139	Homeobox (203–261)	Transcription factor
			Telencephalic development
HOXA1	65-HHHHHHHHHH-74	Homeobox (229–291)	Transcription factor
			Hindbrain segmentation
HOXA9	84-HHHHHH-89	Homeobox (207–267)	Transcription factor
			Positional identity on the anterior/posterior axis
MEOX2*	64-HHRGHHHHHHHHHHHH-79	Homeobox (186–248)	Transcription factor
			Somite development
OTX1*	275-HHHHHPHAHHPLSQSSGHHHHHHHHHH-301	Homeobox (36–96)	Transcription factor
		Otx-box (247–274)	Brain development
HAND1	8-HHHHHHHPHPAH-20	Helix-loop-helix (103–151)	Transcription factor
			Cardiac morphogenesis
CBX4*	380-HHPHPHPHHHHHHHHHHHH-398	Chromodomain (16–69)	Chromatin modification
		CtBP2-interacting domain (470–475)	SUMO E3-ligase
		RING2-interacting domain (540–558)	
FOXG1B	33-HHASHGHHNSHHPQHHHHHHHHHHH-57	Fork-head domain (179–269)	Transcription factor
		PLU-1-interacting domain (375–411)	Regulator of telencephalon morphogenesis
		FAST2-intracting domain (314–372)	
PRICKLE3	513-HHHHNHHHHHNRH-525	PET domain (73–178)	Unknown
		LIM domain (186–243)	
*DLX2*	309-HHHHHHH-315	Homeobox (157–210)	Transcription factor
			Forebrain differentiation
*POU4F1*	100-HHHHHHHHH-108	POU domain (279–291)	Transcription factor
		Homeobox (306–319)	Differentiation and survival of sensory neurons
		Homeobox (389–412)	
*ZIC3*	87-HHHHHHHHHHH-97	Zinc finger (300–322; 328–352; 358–382; 388–410)	Transcription factor
			Determination of left-right asymmetry
*ONECUT1*	124-HHHHHHHHHHHPHH-138	CUT domain (283–369)	Transcription factor
		Homeobox (385–477)	Pancreas specification
		CREB-interacting domain (327–331)	
*MAFA*	184-HHHGAHHAAHHHHAAHHHHHHHHHSHGGAGHGGGAGHH-219	Maf-N (111–145)	Transcription factor
		Basic leucine zipper (253–316)	Regulator of insulin gene expression
*MAFB*	131-HHHHHHHHPHPHHAYPGAGVAHDELGPHAHPHHHHHH-167	Maf_N (80–114)	Transcription factor
		Basic leucine zipper (209–303)	Regulator of lineage-specific haematopoiesis
*MEC2P*	366-HHHHHHH-372	Methyl-CpG-binding domain (94–168)	Transcription repressor

First group (in bold) = proteins that accumulate in nuclear speckles under basal conditions; second group = proteins that accumulate in nuclear speckles after a-amanitin treatment.

*: deletion of DNA-binding/protein-protein interaction motifs was also tested; third group (in italics) = proteins that do not localize in speckles.

The dependence on the polyHis segment for this dynamic behavior was analyzed using a HOXA1 mutant protein in which the His-repeat was eliminated. Accordingly, there was no change in the subcellular distribution of this mutant protein when cells were exposed to α-amanitin (Figure 7C). HOXA1-dependent reporter assays confirmed that deletion of the His-repeat did not abolish the transcriptional activity of this transcription factor (Figure 7D), suggesting that the mutation affected specifically the subnuclear localization of the protein.

We also analyzed the effect of RNA polymerase II inhibition on three polyHis-containing proteins considered to be cytosolic: the negative regulator of the Wnt-canonical pathway NKD2; the mitotic kinase PLK2; and the PRICKLE family member PRICKLE3 (also known as LMO6). Both NKD2 and PLK2 remained in the cytoplasm under basal conditions and upon exposure to α-amanitin (not shown). However, exposure to this inhibitor produced the translocation of a proportion of PRICKLE3 to the nucleus, where it co-localized with SC35 (Figure 8A). Incubation with leptomycin B, an inhibitor of CRM1-dependent nuclear export, caused the relocalization of PRICKLE3 to the nucleus (Figure 8B), indicating that it is a shuttling protein and further suggesting that its targeting to nuclear speckles may be linked to the yet unknown role of PRICKLE3 within the nucleus. Leptomycin B treatment induced accumulation of PRICKLE 3 in PML bodies (Figure S7).

The results of the analysis of the subcellular localization of several polyHis-containing nuclear proteins are summarized in Table 1 and notably, 15 out of 22 of these proteins displayed nuclear staining compatible with their accumulation in nuclear speckles. Thus, proteins with His-repeats seem to localize dynamically in the splicing factor compartment.

## Discussion

SARs are frequently found in eukaryotic proteomes [2],[44]. It has been suggested that their physicochemical properties, such as flexibility or low-affinity interactions, confer certain advantages over other types of amino acidic regions [5]. However, the role of many SARs is unknown and therefore, efforts have been made to perform global surveys of this type of sequence in order to identify common functional features [2],[4],[5]. We have performed an exhaustive analysis of the proteins containing His-tracts in the human genome, confirming that His-repeats are uncommon within proteomes. Moreover, they tend to be well conserved between human and mouse, with about 85% of them showing at most one repeat unit size difference. The low rates of heterozygosity observed in (CAC)n microsatellites in coding regions also suggest that the evolution of these His-repeat has been limited [45]. Although His-tracts of moderate length are likely to have been positively selected in human proteins, as shown by the comparison to CAC/CAT repeats in non-coding regions, there may be a limit to the repeat size. In fact, we noted the absence of pure tracts with more than 15 His-residues, whereas much longer tracts may exist for other SARs. For example, alanine repeats of 25 residues are present in several developmental proteins [13], and non-pathogenic glutamine tracts may reach about 60 repeat units [11]. Size restriction might be linked to the possible pathogenic effects of His-tracts longer than 15 residues.

The presence of multiple SARs is not uncommon in human proteins [2],[4] and polyHis-containing proteins are no exception since a large fraction of them contained alanine, glycine, serine, proline or glutamine SARs. Besides, half of the proteins with His-repeats contained extended tracts interrupted by other amino acids. Interestingly, the most common interrupting amino acids were those that typically form homopeptidic stretches in transcription factors, such as proline, glutamine or glycine. Enrichment of this type of amino acids has also been observed in polyglutamine containing proteins [46]. Stretching this idea further, repeats may often grow within repeats, as illustrated by the appearance of SSS, PPP or GGG repeats within extended His-repeats (Table 1). Moreover, the disrupting residues may act as brakes for the expansion of the pure repeats, and restrict the size of the His-repeat, which in turn might reduce the likelihood of protein aggregation and associated pathogenic effects.

### The His-Repeat Is a Novel Nuclear Speckle-Localization Signal

The mammalian nucleus is a highly complex organelle that is both physically and functionally compartmentalized (for review, see [22],[47]). The subnuclear structures are associated with specific biological activities related to the synthesis, processing and modification of RNA, and they can be distinguished by morphological criteria and the presence of specific protein markers. One such compartment is that of the nuclear speckles. The mechanisms responsible for the formation and regulation of these structures are not yet known and as for many other nuclear bodies, it has been proposed that they are highly dynamic self-organizing entities [48]. A rapid exchange of protein components between subnuclear compartments has been reported, which can be explained by a reaction-diffusion model [49]. However, the kinetics associated to a particular protein can be affected by its binding to other molecules, either proteins or nucleic acids, which in turn can aid its recruitment to a specific compartment. Accordingly, a few protein domains have been described that direct proteins to nuclear speckles, such as the arginine/serine-rich (RS)-domain in SR proteins [50] or the RNA recognition motif [51]. Other regions in specific proteins have also been reported to act as speckle-localizing sequences, like the threonine-proline repeats in SF3B1/SF3b^155^ [52] and the “Forkhead Associated” domain in PPP1R8/NIPP1 [53].

We previously showed that the His-tract in the DYRK1A protein kinase and the regulator of transcription cyclin T1 [20],[21] is responsible for the accumulation of these proteins in nuclear speckles. Given that the functions of many of the polyHis-containing proteins were related to DNA and RNA metabolism, it was plausible that this role as a subnuclear targeting signal could be more general in other proteins. Indeed, a significant proportion of the polyHis-containing proteins analyzed have the ability to accumulate in nuclear speckles either at the steady-state or upon transcription inhibition. This targeting may respond to the nature of nuclear speckles as sites of storage, recycling and degradation of factors involved in DNA and RNA metabolism [22],[54]. The uneven distribution found among different speckle-positive His-repeats-containing proteins is also observed among splicing factors that accumulate in speckles for instance [22],[26], and could reflect differential binding affinities for distinct targets within the nucleus. Importantly, accumulation in nuclear speckles is dependent on the presence of the His-tract, as confirmed by both deletion analysis in some candidate proteins and by the behavior of paralogous proteins lacking the His-repeat. Apart from the previously mentioned DYRK1A and cyclin T1 [20],[21], only HOXA9 had already been reported to accumulate in nuclear speckles of unknown nature [55].

Given that our analysis was performed by transient transfection of plasmids expressing the candidate proteins fused to GFP, we tried to rule out non-physiological effects due to overexpression. This is particularly relevant since expanded homopolymeric tracts, including polyHis expansions, have been associated with protein aggregation [16],[31],[56]. As a cellular defense mechanism against protein misfolding and aggregation, protein aggregates are thought to be sequestered in inclusions that also contain molecular chaperones and components of the ubiquitin proteasome system [57]. We did not detect any co-localization of candidate proteins with an anti-ubiquitin antibody (Figure S8), suggesting that the speckled staining was not produced by the formation of intranuclear protein aggregates. In addition, no cytosolic granules were detected (Figure S1 and S2), in contrast with results published with longer His tracts (26 His residues; [31]). We also analyzed the behavior of a stably expressed polyHis-containing protein (DYRK1A) fused to GFP during the cell cycle. Nuclear speckles disassemble when cells enter mitosis and the proteins associated with them become diffusely distributed throughout the cytoplasm [58]. As shown in Figure S9, the fusion protein totally recapitulated these changes during the cell cycle indicating that poly-His expression does not interfere with the intrinsic dynamics of the compartment. As additional support for the specificity of the subcellular localization, we did not detect an accumulation of the GFP-9xHis chimera in other subnuclear compartments and there was no colocalization with different marker proteins or any specific accumulation in the cytoplasm of the transfected cells, suggesting that the fusion protein is not recruited to a specific cytosolic organelle.

It seems most likely that the His-repeat acts as a nuclear speckle-targeting signal by serving as an interaction surface for resident molecules in the speckle. The features of His make it a versatile amino acid, strongly represented in enzymatic and binding activities. Histidine's imidazole side-chain allows it to shift from a neutral to positive charge in a pH-dependent fashion, a property that may have an impact on the binding capabilities of a His-stretch. Moreover, the presence of His in a β-strand provides a charge gradient that could mediate protein-protein or protein-DNA via electrostatic interactions. His is also known as an excellent ligand to coordinate metal ions [17], which can also participate in organizing interacting domains. All these mechanisms may contribute to finely regulate the binding properties of His-repeats. Examples of His-stretches as protein-protein interacting domains can be found in cyclin T1 when interacting with RNA polymerase II and granulin [18],[19], and DYRK1A interacting with Sprouty2 [59].

The ability of His-tracts to target proteins to the nuclear speckles compartment seems to be specific to His since other homopolymeric amino acid tracts do not display such activity according to our results (9xGln and 9xPro as GFP fusions; 13xAla in NLKΔHis, 16xGly and 7xSer in POU4F2ΔHis) and those published for longer amino acid tracts [31]. Speckle-positive His-repeats vary from simple amino acid runs (for instance, **H_10_** in HOXA1) to complex repeats (**H**PSN**H_5_**N**H_2_**S**H**K**H**S**H** in cyclin T1), suggesting that the number of His residues is not decisive for its functional role but rather, the spacing between residues may be important. We failed to find a specific code underlying targeting to nuclear speckles, except that a minimum of 6 His residues is required for this effect, which indicates a high degree of flexibility in this functional signal. Considering that His-repeats are widely used as tags for affinity-purification and immunodetection of expressed proteins, we would like to stress the fact that more than 6 His residues may alter the original localization of a tagged protein.

### His-Repeats as a Way of Generating Evolutionary Diversification in Gene Duplicates

Only 22% of SARPs have paralogous proteins [60], whereas a large fraction of the genes encoding proteins with His-repeats have closely-related paralogues. We found that many of them were derived from gene duplications at the base of vertebrate evolution, when two rounds of whole-genome duplication took place [61]. Interestingly, most of the paralogues lacked the His-repeat, suggesting that this repeat had been gained after the duplication of the gene. Further analysis of the distribution of these repeats revealed that they were gained during two periods of vertebrate evolution: soon after gene duplication or before placental mammal radiation.

The comparison of the subcellular distribution of three pairs of paralogous proteins, FAM76B/FAM76A, DYRK1A/DYRK1B and POU4F2/POU4F3, confirmed that only those members containing His-repeats localized to nuclear speckles. Notably, in approximately 30% of the duplicate gene pairs derived from the *S. cerevisiae* whole-genome duplication event, the two protein members of the pair localize to distinct subcellular compartments [62]. This and other evidence led to the proposal that protein subcellular relocalization might be an important evolutionary mechanism for the functional diversification of duplicate genes [63]. Therefore, the appearance of a new repeat, or variations in the length and composition of an existing one, may have been an important mechanism for functional diversification. The acquisition of a new His-repeat might have contributed to the reorganization of protein-protein interaction networks and more specifically, to nuclear speckle targeting as a novel cell property associated to the paralogous protein. This might be relatively rapid on an evolutionary time scale because of the high mutation rates associated with microsatellites [64]. In fact, the expansion and contraction of repeats within transcription factors has been linked to major morphological changes in vertebrates [65],[66]. Given that a high proportion of the polyHis-containing proteins have roles in developmental processes, mutations involving His-repeats may have played a significant part in diversification and adaptation.

### Subnuclear Localization of PolyHis-Containing Proteins Is a Highly Dynamic Process

Several of the His-containing proteins that did not accumulate in nuclear speckles were transcription factors. The fact that these proteins contain domains that may control their specific localization within the nucleus, such as DNA binding regions or protein-protein interaction domains, led us to think that competition between His-repeats and other protein regions might regulate their intranuclear distribution. Our results show a direct correlation between loss of DNA binding activity and accumulation in nuclear speckles. Similar behavior was recently described for the transcription factor GATA-4, although the subnuclear compartment to which it localized was not identified [67]. Although we cannot ignore that the elimination of the DNA binding domains may result in a conformational change that exposes the His-repeat, we favor a loss of retention in the chromosomal compartment as being responsible for the enrichment in nuclear speckles. This assumption is supported by the results with inhibitors of RNA polymerase II-dependent transcription, since treatment with α-amanitin caused re-localization to nuclear speckles of many of the proteins with a dispersed nuclear distribution under basal conditions. In this regard, we noted that the subgroup of proteins unable to accumulate in nuclear speckles was enriched in proteins with more than one DNA binding domain, a feature that may confer a more immobile character to these proteins. Thus, we propose that the intranuclear localization of some transcription factors with His-repeats is the net result of competition for binding to different recruiting sites within the nucleus, such as DNA, nuclear speckles or other nuclear bodies. Moreover, this dynamic behavior might also explain why among the proteins listed in Table 1, only OTX1 appeared in a proteomic analysis of enriched preparations of interchromatin granule clusters [24]. Such a proteomic analysis would not consider proteins present in low amounts and/or proteins that are transiently found in such structures.

It is widely accepted that RNA processing occurs co-transcriptionally and thus, there is a co-localization of factors related to RNA biogenesis, such as transcription and splicing factors [68]. When needed, transcription factors are recruited to specific promoters in active transcription sites whereas splicing factors are assembled into the spliceosome. During transcriptionally inactive periods, the splicing factors re-locate to the speckle domains, and some transcription factors might also behave similarly. Transit through the speckles may provide the opportunity for transcription factors to encounter RNA processing factors and/or other transcription factors, and to assemble into complexes acting on the same gene. This re-localization may also involve the targeting of transcription factors no longer able to bind DNA to other compartments for degradation or other processing activities 54,69. In addition, compartmentalization of transcription-related proteins within distinct nuclear bodies may be an important mechanism to regulate gene expression. For instance, the inactivation of the transcription factor HAND1 by nucleoli retention has been implicated in trophoblast stem cell proliferation and renewal [70], and estrogen receptor-enhanced transcription requires interchromosomal interactions at nuclear speckles [71]. The presence of a common sequence to direct a subset of proteins to nuclear speckles, such as the His-repeats, may confer functional advantages. First, it may represent a way to concentrate functionally related proteins, perhaps facilitating their physical interaction. Second, it may reflect a common mechanism to regulate these proteins. Indeed, given that most of the polyHis-containing proteins are involved in developmental processes, His-repeats may be a means of keeping transcription factors away from promoters when they are not required.

Uncontrolled expansion of SARs is associated with developmental and neurodegenerative human diseases (for review, see [2],[11],[13]), although no pathological His expansions/deletions have yet been reported. However, variants in the length of the His-repeats in the HOXA1 protein have been described in the Japanese population [56], and the expression of these variants compromised HOXA1 function in neuronal differentiation [72]. Furthermore, a polyHis polymorphism in *ZIC2* is apparently associated with neural tube defects [73]. Intriguingly, no homozygous cases of expansions have been found in either of these genes. On the basis of these data, and considering that some polyHis-containing proteins are fundamental for essential developmental processes, variation in His-repeats would be expected to contribute to human disease.

## Materials and Methods

### Genome-Wide Computational Search for His-Repeats

An in-house Perl computer program was used to identify all human proteins containing a tandem His-repeat of 5 residues or more from Ensembl (version 48, http://www.ensembl.org/, [32]). When more than one protein per gene existed, we selected the longest of these. One to one orthologous proteins from mouse, as well as human paralogous genes, were identified using Ensembl Biomart annotations. The paralogous gene analysis was restricted to those genes derived from duplication events at the Euteleostome or more recent levels, since these homologues were sufficiently similar to produce reliable alignments. The procedure used to map equivalent repeats in two homologous sequences has already been described [82]. Briefly, for each repeat found in a sequence, we determined whether an equivalent repeat existed in the orthologous sequence by looking for His-repeats that overlapped with the reference repeat in the pairwise protein sequence alignment available from Ensembl. Non-coding tandem CAY (CAC/CAT) repeats were recovered from the non-protein coding parts of the genome (goldenpath 200603).

### Gene Ontology-Based Analysis of Protein Function

We obtained all available Gene Ontology annotations (GO, http://www.geneontology.org/, [33]) for human genes from Ensembl (18,086 genes). The number of genes annotated with the terms ‘nucleus’, ‘cytoplasm’ (excluding those also annotated as ‘nucleus’) and ‘membrane’ (excluding those also annotated as ‘nucleus’ and/or ‘cytoplasm’) in the cellular compartment classification were counted. In the complete dataset, 4634 genes were annotated as ‘nucleus’, 191 as ‘cytoplasm’ and 4257 as ‘membrane’. Out of 82 polyHis-containing proteins with GO annotations, 59 were annotated as ‘nucleus’, 2 as ‘cytoplasm’ and 7 as ‘membrane’. Several terms related to transcriptional regulation and to developmental processes were particularly abundant among the proteins with His-repeats. To avoid redundancy in the functional analysis, three groups of nuclear proteins were selected: 1) genes with GO annotations related to nervous system development (‘nervous system development’, ‘central nervous system development’, ‘brain development’, ‘hindbrain development’, ‘forebrain development’, ‘midbrain development’ and ‘dendrite development’); 2) genes with GO annotations related to other developmental processes (terms containing ‘development’ not included in the previous class); and 3) genes with the GO annotation ‘regulation of transcription’ (and not included in the two previous classes). In the complete dataset, 142 genes were included in the first class, 585 in the second class and 1829 in the third. Among polyHis-containing genes, 13 genes were included in the first class, 12 in the second class and 19 in the third class.

### Statistical Analysis

To detect any statistical differences in the distribution of the repeat sizes we used the non-parametric Kolmogorov-Smirnov test. To detect over-represented GO terms we used the binomial probability. Statistical analyses were performed with the R statistical package (http://www.r-project.org/).

### Plasmids

The expression plasmids encoding GFP-tagged human DYRK1A (754 amino acid isoform; pGFP-DYRK1A) has been described [20]. The plasmid expressing GFP fused to the DYRK1A fragment 378–616 (H+) was obtained by in-frame subcloning of the appropriate PCR fragment into pEGFP-C1 (Clontech). Expression plasmids for DYRK1B (pGFP-DYRK1B, [74], SC35 (pYFP-SC35, [75], POU4F1 (pTS-Brn3a, [76], cyclin T1 (pMyc-Cyclin T1, [19], NKD2 (pGFP-NKD2, [77], and CBX4 (pGFP-CBX4) were kindly provided by W. Becker (Aachen University, Germany), D. Spector (Cold Spring Harbor Laboratory, Cold Spring Harbor, USA), E. Turner (Department of Psychiatry, University of California, USA), M. Peterlin (Howard Hughes Medical Institute, University of California, USA), C. Li (Department of Medicine, Vanderbilt University Medical Center, USA), and S. Aznar-Benitah (Centre for Genomic Regulation-CRG, Spain), respectively.

To generate the plasmids expressing the different GFP fusion proteins, the corresponding open reading frames were PCR amplified with specific primers using IMAGE Consortium cDNA clones as templates (http://image.llnl.gov/, [78]). The identification number of the IMAGE clones and the sequence of the primers used are listed in Table S3. All the IMAGE clones were purchased from the RZPD German Resource Center for Genome Research. Details of the generation of all constructs used in this study are provided in the Supporting Materials and Methods (Text S1). Plasmid pG4-HOXA1 was constructed by fusing the nucleotide sequence corresponding to the HOXA1 open reading frame in-frame with the yeast Gal4 DNA binding domain (DBD) in pG4-DBD [79]. To obtain plasmids expressing 5xHis, 6xHis, 7xHis, 8xHis and 9xHis or 9xPro and 9xGln protein segments fused to GFP, double stranded oligonucleotides (Table S4) were annealed and ligated into the *Bgl*II and *EcoR*I sites of the pEGFP-C1 expression vector. Deletion of His-repeats was performed by site-directed mutagenesis (Stratagene) on pHA-DYRK1A, pFlag-POU4F2, pGFP-NLK, pGFP-HOXA1 and pG4-HOXA1. All plasmids generated by PCR, as well as all the in-frame fusions, were verified by DNA sequencing.

### Cell Culture and Transfection

The U2-OS, HeLa, CV-1 and HEK-293 cell lines were maintained at 37°C in Dulbecco's Modified Eagle's Medium supplemented with 10% fetal calf serum (FCS) and antibiotics. Transient transfections were performed using the calcium phosphate precipitation method and the cells were processed 24–48 h after transfection. For the generation of stable cell lines, transfected U2-OS cells were selected by incubation with G418 (500 µg/ml; Gibco-Invitrogen) for 10 days and the clones derived from a single cell were isolated. Cell lines were maintained in G418 (250 µg/ml). Treatment of HeLa cells with RNA polymerase II inhibitor, α-amanitin (50 µg/ml; Sigma) and with the CRM1-dependent export inhibitor leptomycin B (10 ng/ml; Sigma) was carried out for 5 h at 37°C.

### Immunofluorescence

HeLa cells (7×10^5^) growing on coverslips in six-well dishes were transfected with the different expression constructs and 48 h after transfection, the coverslips were washed in cold phosphate buffered saline (PBS), fixed in 4% paraformaldehyde in PBS for 15 min, and permeabilized in 0.1% Triton X-100 in PBS for 10 min. For ubiquitin detection, the cells were fixed in methanol for 2 min at −20°C, and they were then blocked with PBS-10% FCS for 30 min and incubated with primary antibodies for 1 h at room temperature. After washing extensively with PBS-1% FCS, the coverslips were incubated with the secondary antibodies for 45 min at room temperature, washed repeatedly with PBS-1% FCS, and mounted onto slides using Vectashield Mounting Medium (Vector Laboratories) plus 0.2 µg/ml 4′,6-diamidino-2-phenylindole (DAPI) or TO-PRO-3 (Molecular Probes). Images were acquired with an inverted Leica SP2 Confocal Microscope and GFP was excited with the 488 nm line of the Argon laser while IgG Alexa 647 was excited with a 633 nm HeNe laser. The following antibodies were used as primary antibodies: monoclonal anti-SC35 antibody (BD Pharmigen, 1∶100), monoclonal anti-ubiquitin antibody (P4D1, Santa Cruz Biotechnology, 1∶50), rabbit polyclonal anti-DYRK1A antiserum ([80] 1∶250), rabbit polyclonal anti-PML antiserum (Santa Cruz Biotechnology, 1∶100), mouse monoclonal anti-SUMO1 antibody (Santa Cruz Biotechnology, 1∶100), rabbit anti-PSP1 antiserum (Dundee Cell Products, 1∶500), rabbit polyclonal anti-S1-1 antiserum (a kind gift of Dr. A. Inoue, [Osaka City University Graduate School of Medicine, Osaka, Japan]; [43]) and goat polyclonal anti-POU4F2 antiserum (Santa Cruz Biotechnology, 1∶1000). The secondary antibodies used were an Alexa 647-conjugated goat anti-mouse (Molecular Probes, 1∶400), an Alexa 555-conjugated donkey anti-mouse (Invitrogene, 1∶400), an Alexa 488-conjugated donkey anti-goat (Molecular Probes, 1∶400), an Alexa 555-conjugated goat anti-rabbit (Molecular Probes, 1∶400) and fluorescein isothiocyanate conjugated goat anti-rabbit (Southern Biotechnology, 1∶400).

### *In Vitro* Kinase Assays

Transfected HEK-293 cells (2×10^6^) were lysed in Hepes lysis buffer (50 mM Hepes pH 7.4, 150 mM NaCl, 1% NP-40, 2 mM EDTA, 2 mM NaVO_4_, 30 mM NaPPi, 25 mM NaF) supplemented with a cocktail of protease inhibitors (Roche). Soluble extracts were immunoprecipitated either with anti-HA (Abnova) or anti-GFP (Molecular Probes) antibodies. Immunocomplexes were washed twice with kinase buffer (50 mM Hepes pH 7.4, 5 mM MgCl_2_, 5 mM MnCl_2_, 0.5 mM DTT) and incubated in 30 µl of kinase buffer with 10 µM ATP and [g^32^P]-ATP (6.5×10^−3^ µCi/pmol) for 20 min at 30°C. For DYRK1A, kinase activity was followed by phosphate incorporation on the synthetic peptide DYRKtide (200 µM) in a liquid scintillation B-counter (Beckman Coulter) as described previously [80]. For NLK, the reaction was stopped by adding 2× loading sample buffer and the samples were resolved by SDS-PAGE. ^32^P incorporation was detected by autoradiography of the dried gels.

### Reporter Assays

For the POU4F2-dependent reporter assay, CV-1 cells (1×10^5^) were seeded in 35-mm dishes. The cells were transfected with a luciferase reporter plasmid driven by the minimal prolactin promoter plus 3 repeats of the POU4 family recognition site (pGL2-3xBrn3a, kindly provided by E. Turner; [81]) together with pFlag-POU4F2 or pFlag-POU4F2ΔHis and a β-galactosidase expressing plasmid as an internal control. For HOXA1-dependent reporter assays, cells were transfected with the pG5E1B-luc reporter (luciferase is driven by five repeats of the synthetic Gal4-binding sites introduced upstream of the minimal adenovirus E1b promoter; [79]) together with pG4-HOXA1 or pG4DBD-HOXA1ΔHis encoding chimeras of HOXA1 proteins fused at their N termini to the Gal4 DBD. A *Renilla* luciferase plasmid (pCMV-RNL, Promega) was used as an internal control. Cells were lysed 48 h post-transfection and the activity of both luciferase enzymes was measured with the Dual-Luciferase Reporter Assay kit (Promega). Each transfection was carried out in triplicate.

## Supporting Information

Figure S1 The ability of a His-tract to direct a heterologous protein to the nuclear speckles depends on the number of consecutive His residues. A) HeLa cells were transfected with expression plasmids encoding GFP fusion proteins with different numbers of His residues: 5xHis, 6xHis, 7xHis, 8xHis or 9xHis repeats. The localization of the fusion proteins was analyzed by direct fluorescence (left column, green) and by immunofluorescence for SC35 (middle column, red). Merged images are also shown (left column). The unfused GFP protein was used as a control and co-localization with the endogenous marker was determined by confocal imaging. B) Using the lines on the merged image for GFP-9xHis, fluorescence intensity profiles were obtained for GFP (green) and SC35 (red).
(1.26 MB PDF)

Figure S2 His homopolymeric tracts specifically target proteins to the nuclear speckle compartment but not to other nuclear bodies. HeLa cells were transfected with an expression plasmid encoding a GFP fusion protein of with 9xHis residues. The localization of the fusion protein was analyzed by direct fluorescence (left column, green) and by indirect immunofluorescence for markers of different nuclear bodies as indicated (middle column, red). Merged images are also shown (left column). Co-localization with the endogenous markers was determined by confocal imaging.
(1.72 MB PDF)

Figure S3 Deletion of the His-tract in NLK interferes with NLK subnuclear localization but not with its kinase activity. A) HeLa cells were transfected with the expression plasmids for the fusion proteins GFP-NLK or GFP-NLKΔHis. Cells were immunostained for SC35 to visualize the nuclear speckles (middle column, red) and GFP fusion proteins were visualized directly by fluorescence microscopy (left column, green). Merged images are shown (right column). Note the lack of accumulation in nuclear speckles of the NLK mutant protein. B) Soluble extracts from cells expressing unfused GFP, GFP-NLK or GFP-NLKΔHis were immunoprecipitated with anti-GFP and assayed in an in vitro kinase assay. The samples were analyzed in Western blots with anti-GFP and autophosphorylation was assessed by autoradiography of the dried gels. The position of marker proteins (in kDa) is indicated. The NLKΔHis mutant version showed no differences in autophosphorylation activity when compared with the wild type protein.
(0.79 MB PDF)

Figure S4 The localization of other pairs of paralogous proteins confirms that the His repeat is necessary for accumulation in nuclear speckles. HeLa cells were transfected with plasmids expressing the GFP fusions of the DYRK family of protein kinases, DYRK1A and DYRK1B (A), and of the POU family of transcription factors, POU4F2 and POU4F3 (B). A schematic representation of each pair of paralogues is presented. (A) NLS: nuclear localization signal; Kinase: kinase domain; PEST: PEST sequences; His: histidine repeat; Ser: serine-rich region. (B) Gly/Ser: segment rich in glycine and serine; UHD: upstream homology domain in POU family members; POUsd: POU specific domain; POUhd: POU homeodomain. The localization of the fusion proteins was assessed by direct fluorescence (left panels) and their accumulation in speckles by co-localization with SC35 (right panels).
(1.15 MB PDF)

Figure S5 The accumulation of some transcription factors with polyHis stretches in nuclear speckles depends on their interaction with DNA. HeLa cells were transfected with the expression plasmids for wild type GFP-OTX1 or GFP-OTX1ΔHB as indicated (see scheme: His: His repeat; NLS: nuclear localization signal; HoBox: homeobox domain; OtxB: Otx box). The subcellular localization of both proteins was analyzed by direct fluorescence (left column, green) and their accumulation in nuclear speckles by immunofluorescence for SC35 (middle column, red).
(0.76 MB PDF)

Figure S6 Inhibiting transcription with α-amanitin forces some His-containing transcription factors to be retained in nuclear speckles. A) HeLa cells were transfected with the expression plasmid encoding the transcription factor HOXA9. At 48 h post-transfection, the cells were treated with α-amanitin to inhibit transcription and immunostained for SC35 to assess the accumulation of both proteins in the SFC compartment (right panels). Nuclear speckles appear larger and rounder as a consequence of the treatment with the inhibitor. Note that co-localization with nuclear speckles was only observed in cells treated with α-amanitin. B) HeLa cells were transfected with pGFP-HOXA1, and double stained for S1-1 (blue) and SC35 (red). Arrows indicate some of the overlapping structures with S1-1 staining and asterisks those with SC35 staining. Images were acquired by confocal microscopy.
(1.12 MB PDF)

Figure S7 Inhibiting export with leptomycin B forces PRICKLE3 to be retained in PML bodies. HeLa cells were transfected with the expression plasmid encoding PRICKLE3. At 48 h post-transfection, cells were treated with leptomycin B for the times indicated to inhibit nuclear export, and immunostained for PML to assess accumulation in PML bodies (right panels). PML bodies appear larger and rounder as a consequence of the treatment with the inhibitor. Note that PRICKLE3 translocates to the nucleus and co-localizes with PML bodies in cells treated with leptomycin B. This behavior in response to leptomycin treatment has been also described for other proteins accumulating in the nuclear speckles compartment, such as the spliceosome component U1A or the transcription factor ZBP1.
(0.87 MB PDF)

Figure S8 The dot-like staining of polyHis-containing proteins does not overlap with ubiquitin-enriched nuclear aggregates. HeLa cells were transfected with the expression plasmid for the fusion protein GFP-DYRK1A, and cells were immunostained for ubiquitin and then analyzed by direct fluorescence (left panel, green) and by immunofluorescence (middle panel, red). A merged image is also shown (right panel). Note that no co-localization of the DYRK1A nuclear speckles with ubiquitin was detected.
(0.30 MB PDF)

Figure S9 A protein with polyHis-stretches mimics the behavior of a component of endogenous nuclear speckles during the cell cycle. An U2-OS stable cell line expressing GFP fused to a fragment of the DYRK1A protein kinase (amino acids 378–616) that contains the polyHis segment was generated and the co-localization of the GFP signal with SC35 was confirmed (data not shown). Cells grown on coverslips were analyzed by direct fluorescence (central panel) and DNA was stained with TO-PRO-3 to distinguish interphase from mitotic nuclei (left panel). Note that GFP-DYRK1A(378–616) is expressed in discrete foci compatible with nuclear speckles in interphase nuclei, whereas during mitosis (prophase, upper panel; anaphase, lower panel) diffuse staining throughout the cytoplasm is observed as a consequence of nuclear speckle disassembly. This behavior mirrors that of endogenous SC35, whose speckled distribution is lost during prophase.
(0.96 MB PDF)

Table S1 Results of the bioinformatics screen used to identify human proteins containing at least one His-repeat of 5 or more residues.
(0.04 MB XLS)

Table S2 Results of the analysis to identify the paralogues of the genes encoding proteins with His-repeats in the human genome.
(0.04 MB XLS)

Table S3 Information on IMAGE clones and the oligonucleotides used to generate all the expression vectors.
(0.06 MB PDF)

Table S4 Oligonucleotides used to obtain the plasmids expressing His-tracts fused to GFP.
(0.05 MB PDF)

Text S1 Supporting Materials and Methods.
(0.07 MB PDF)

Video S1 *In vivo* imaging of GFP-HOXA1. For live cell observations, HeLa cells (7×105) growing on 35-mm MatTek plates (MatTek Corporation) were transfected with the expression plasmids for the GFP-HOXA1 (1 µg) and YFP-SC35 (250 ng) fusion proteins, and 16 h after transfection the cells were transferred to an environmental control box (EMBLEM Technology Transfer) mounted onto the stage of an inverted Leica TCS SP5 confocal microscope. GFP was excited with the 488 nm line and YFP with the 514 line of the Argon laser. The cells were treated with α-amanitin (50 mg/ml) for 5 h at 37°C, and time-lapse images were acquired at 63× every 5 min and processed with the LAS (Leica Application Suite) AF software. Images for GFP-HOXA1 are shown in the Supporting Video S1. The progressive enlargement of YFP-SC35 signals serves as a control of the treatment (Supporting Video S2).
(0.97 MB MOV)

Video S2 *In vivo* imaging of YFP-SC35.
(0.20 MB MOV)
