# Supplementary figures and images for: Genome-Wide Analysis of Histidine Repeats Reveals Their Role in the Localization of Human Proteins to the Nuclear Speckles Compartment
Source: PLoS Genet. 2009 Mar 6;5(3):e1000397. doi: 10.1371/journal.pgen.1000397 (PMC2644819; doi:10.1371/journal.pgen.1000397)

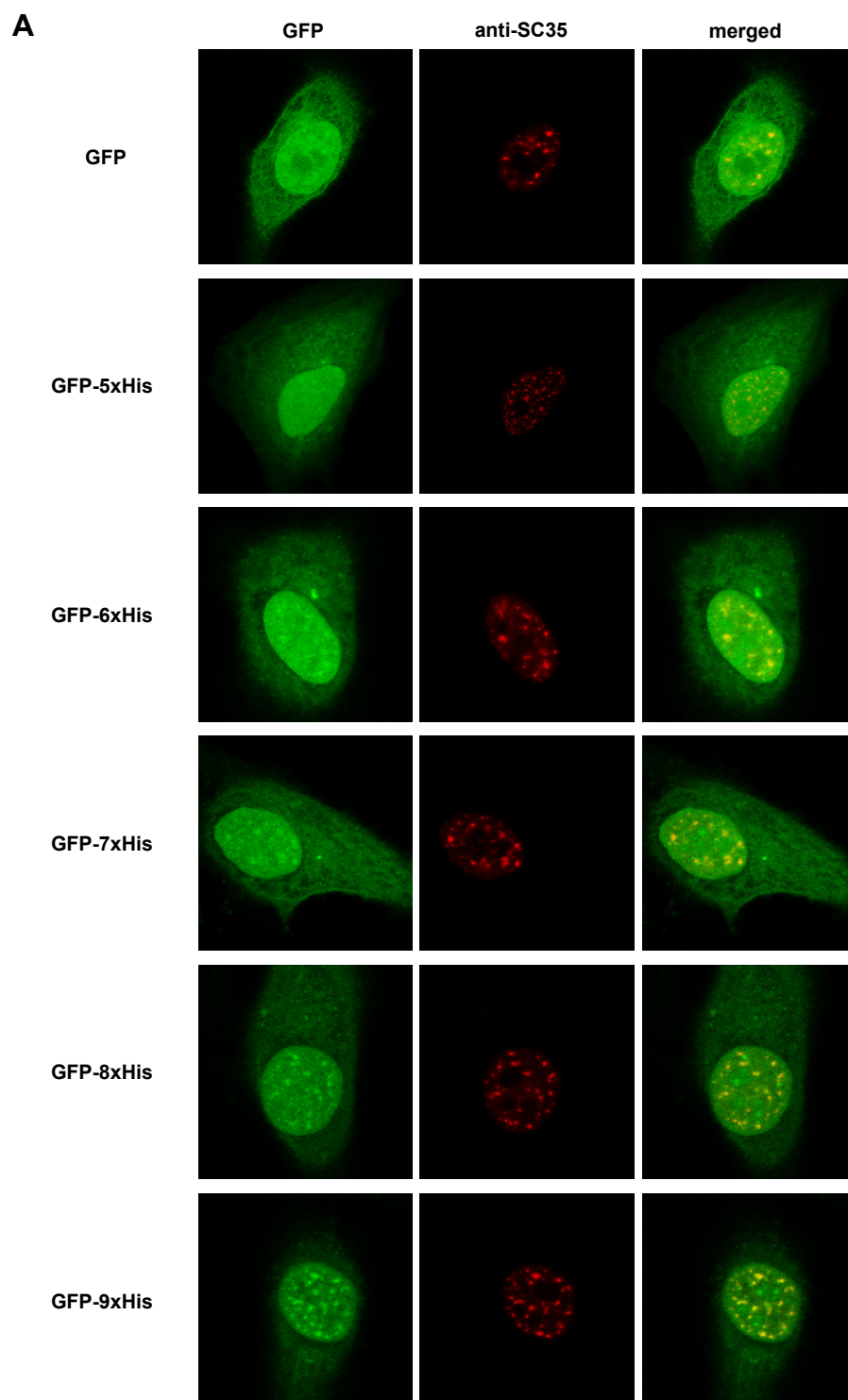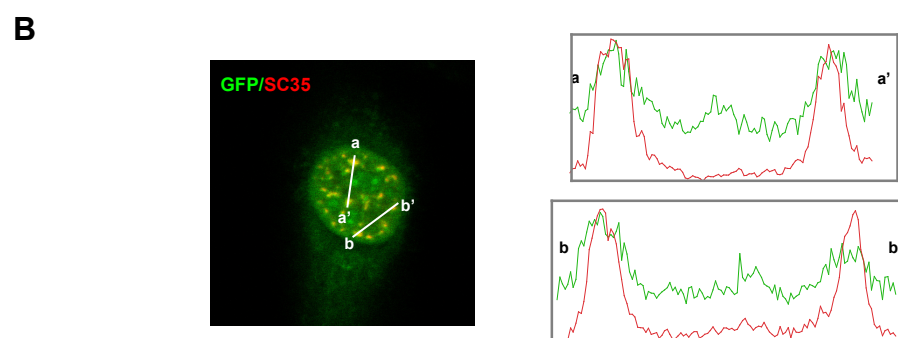

Supplement: Figure S1 — The ability of a His-tract to direct a heterologous protein to the nuclear speckles depends on the number of consecutive His residues. A) HeLa cells were transfected with expression plasmids encoding GFP fusion proteins with different numbers of His residues: 5xHis, 6xHis, 7xHis, 8xHis or 9xHis repeats. The localization of the fusion proteins was analyzed by direct fluorescence (left column, green) and by immunofluorescence for SC35 (middle column, red). Merged images are also shown (left column). The unfused GFP protein was used as a control and co-localization with the endogenous marker was determined by confocal imaging. B) Using the lines on the merged image for GFP-9xHis, fluorescence intensity profiles were obtained for GFP (green) and SC35 (red). (1.26 MB PDF) [file pgen.1000397.s001.pdf]

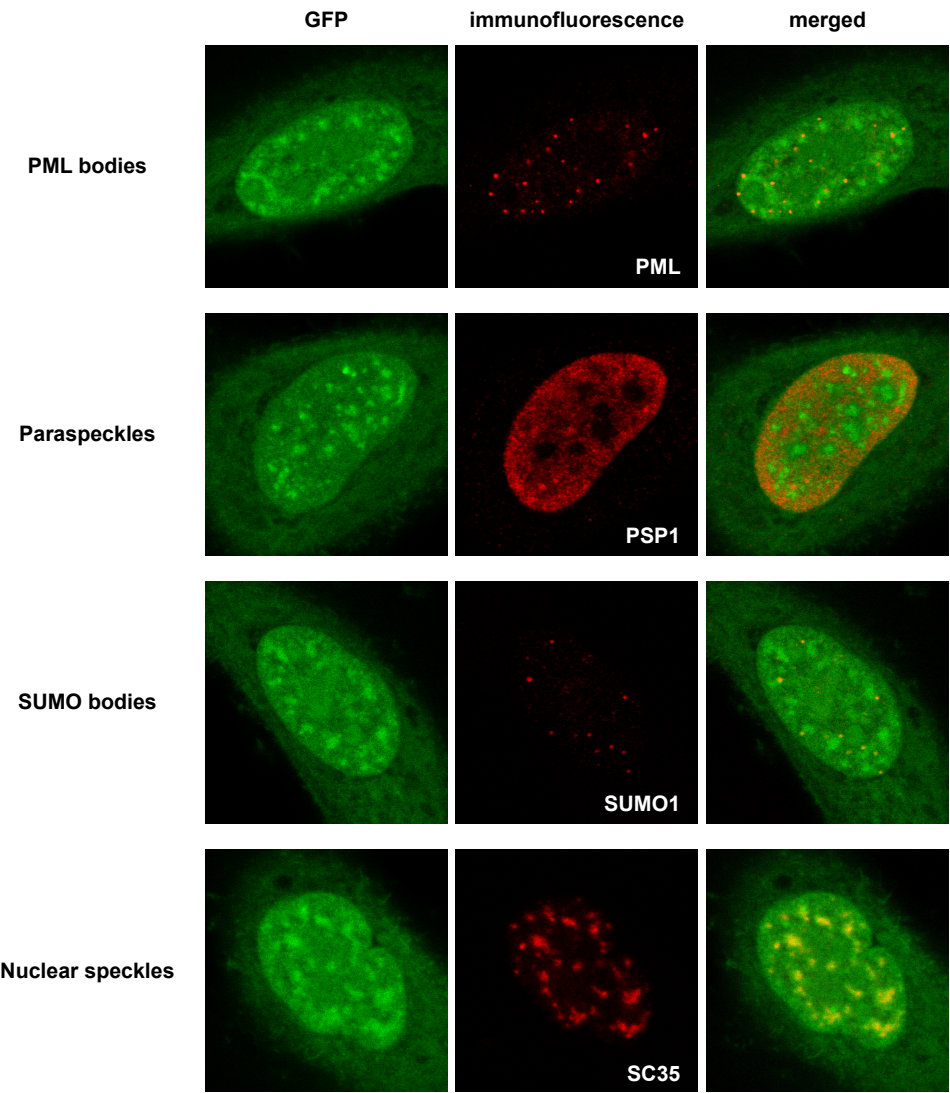

Supplement: Figure S2 — His homopolymeric tracts specifically target proteins to the nuclear speckle compartment but not to other nuclear bodies. HeLa cells were transfected with an expression plasmid encoding a GFP fusion protein of with 9xHis residues. The localization of the fusion protein was analyzed by direct fluorescence (left column, green) and by indirect immunofluorescence for markers of different nuclear bodies as indicated (middle column, red). Merged images are also shown (left column). Co-localization with the endogenous markers was determined by confocal imaging. (1.72 MB PDF) [file pgen.1000397.s002.pdf]

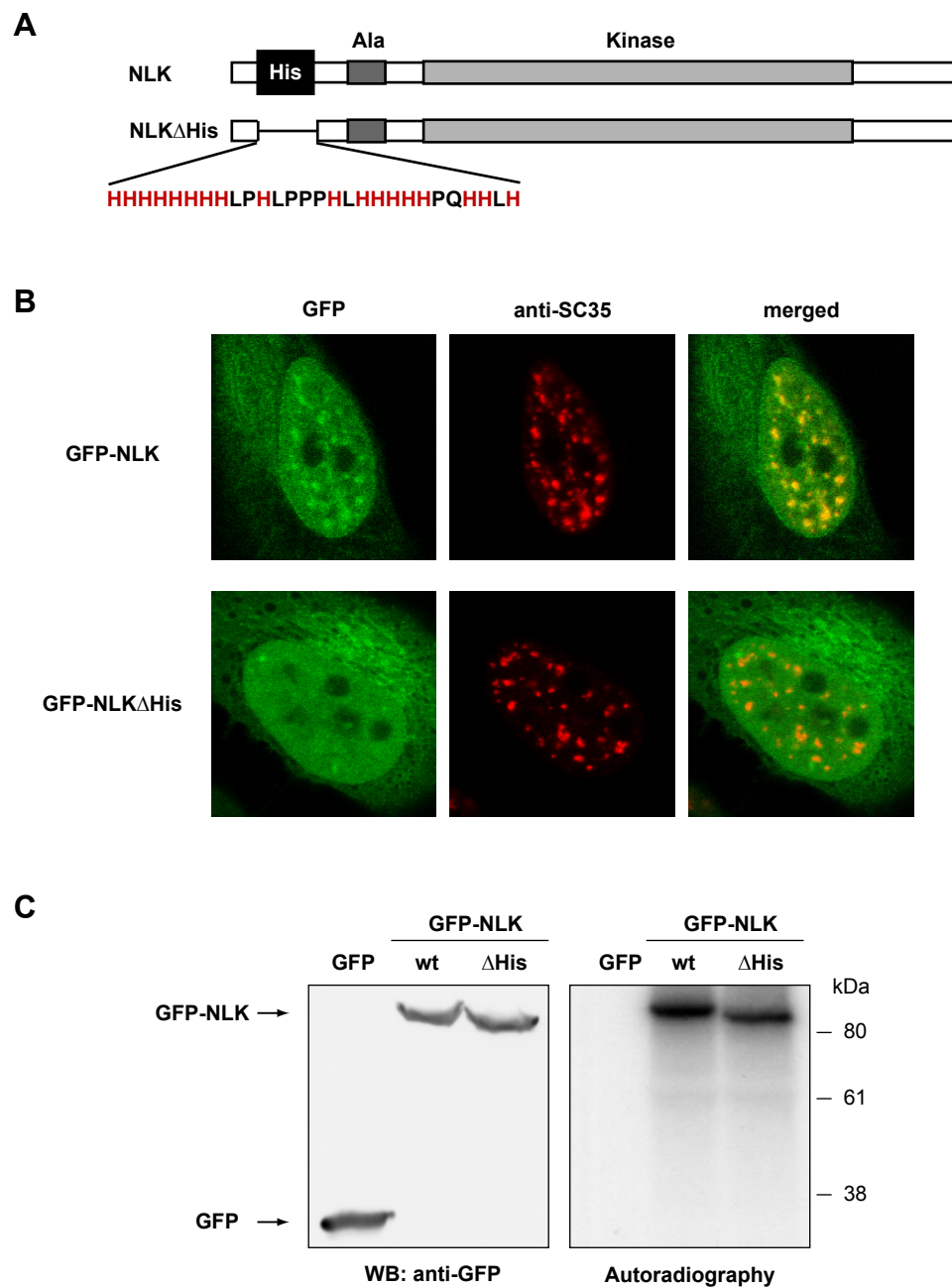

Supplement: Figure S3 — Deletion of the His-tract in NLK interferes with NLK subnuclear localization but not with its kinase activity. A) HeLa cells were transfected with the expression plasmids for the fusion proteins GFP-NLK or GFP-NLKΔHis. Cells were immunostained for SC35 to visualize the nuclear speckles (middle column, red) and GFP fusion proteins were visualized directly by fluorescence microscopy (left column, green). Merged images are shown (right column). Note the lack of accumulation in nuclear speckles of the NLK mutant protein. B) Soluble extracts from cells expressing unfused GFP, GFP-NLK or GFP-NLKΔHis were immunoprecipitated with anti-GFP and assayed in an in vitro kinase assay. The samples were analyzed in Western blots with anti-GFP and autophosphorylation was assessed by autoradiography of the dried gels. The position of marker proteins (in kDa) is indicated. The NLKΔHis mutant version showed no differences in autophosphorylation activity when compared with the wild type protein. (0.79 MB PDF) [file pgen.1000397.s003.pdf]

A

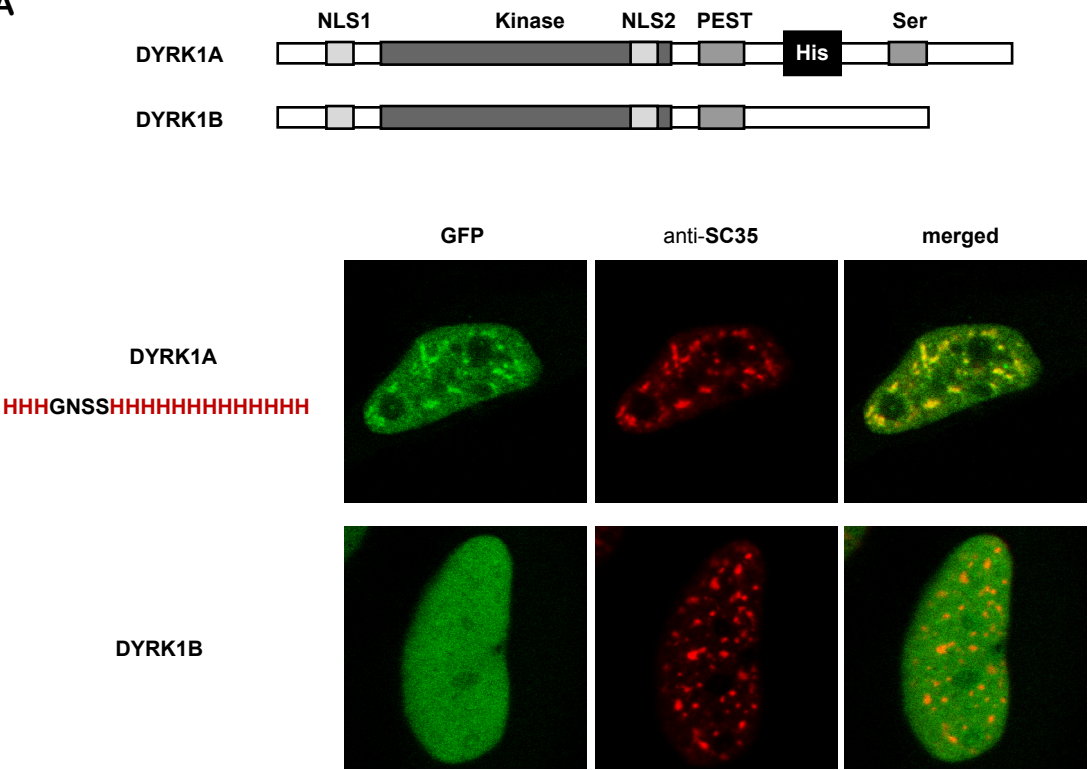

B

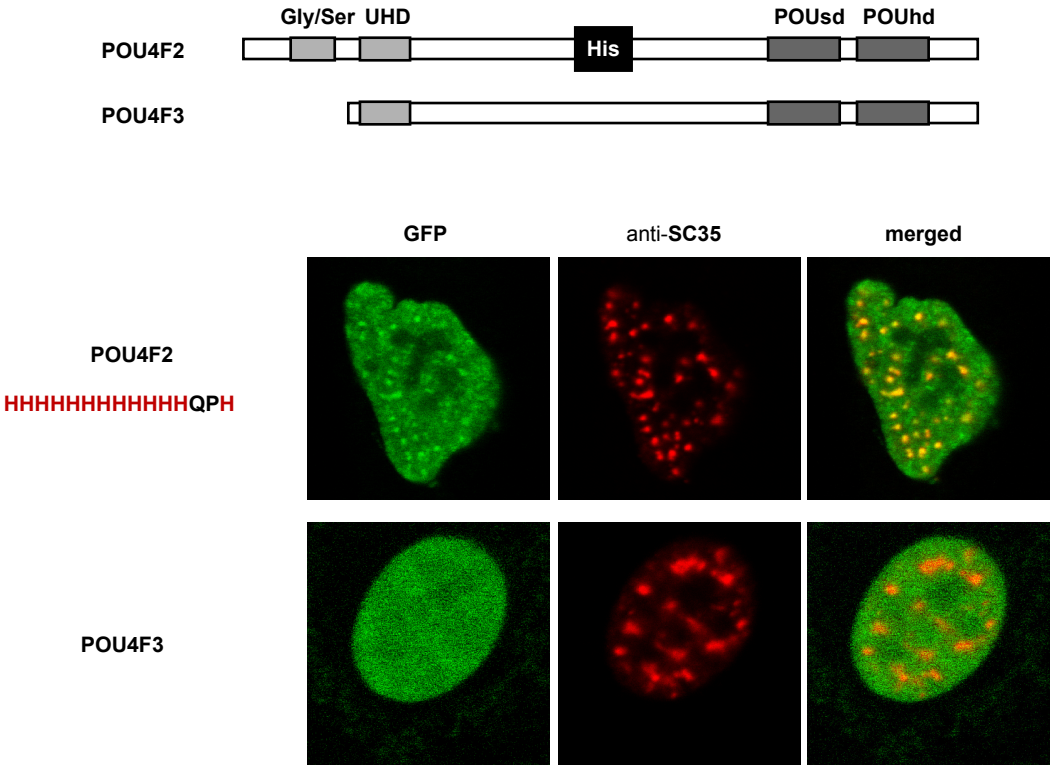

Supplement: Figure S4 — The localization of other pairs of paralogous proteins confirms that the His repeat is necessary for accumulation in nuclear speckles. HeLa cells were transfected with plasmids expressing the GFP fusions of the DYRK family of protein kinases, DYRK1A and DYRK1B (A), and of the POU family of transcription factors, POU4F2 and POU4F3 (B). A schematic representation of each pair of paralogues is presented. (A) NLS: nuclear localization signal; Kinase: kinase domain; PEST: PEST sequences; His: histidine repeat; Ser: serine-rich region. (B) Gly/Ser: segment rich in glycine and serine; UHD: upstream homology domain in POU family members; POUsd: POU specific domain; POUhd: POU homeodomain. The localization of the fusion proteins was assessed by direct fluorescence (left panels) and their accumulation in speckles by co-localization with SC35 (right panels). (1.15 MB PDF) [file pgen.1000397.s004.pdf]

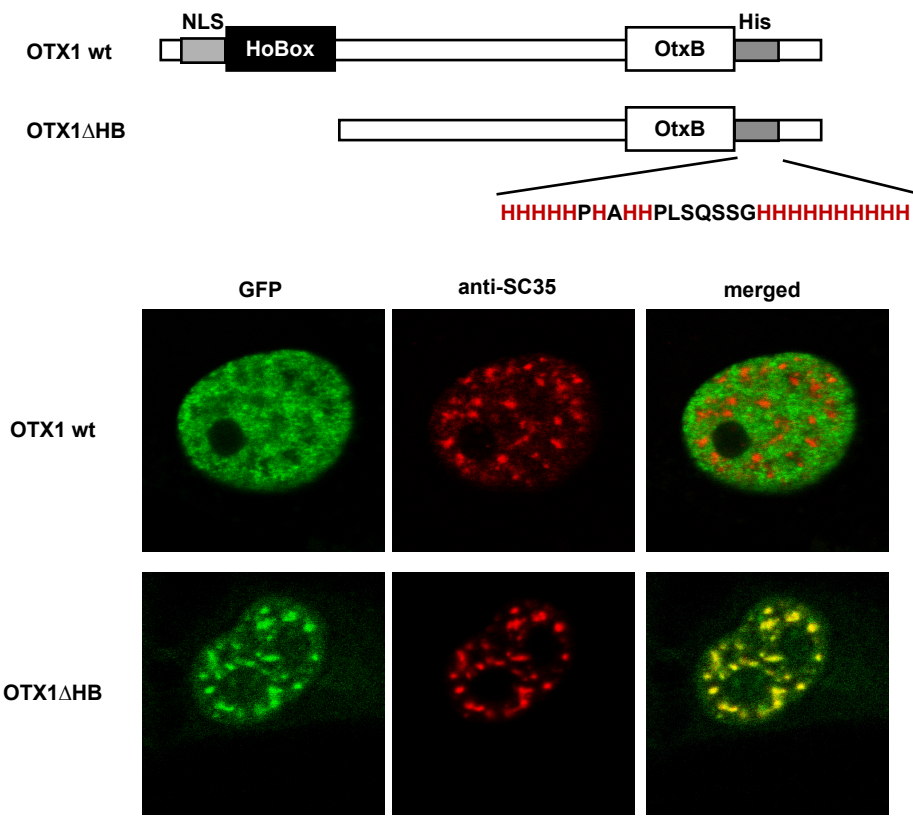

Supplement: Figure S5 — The accumulation of some transcription factors with polyHis stretches in nuclear speckles depends on their interaction with DNA. HeLa cells were transfected with the expression plasmids for wild type GFP-OTX1 or GFP-OTX1ΔHB as indicated (see scheme: His: His repeat; NLS: nuclear localization signal; HoBox: homeobox domain; OtxB: Otx box). The subcellular localization of both proteins was analyzed by direct fluorescence (left column, green) and their accumulation in nuclear speckles by immunofluorescence for SC35 (middle column, red). (0.76 MB PDF) [file pgen.1000397.s005.pdf]

**A**

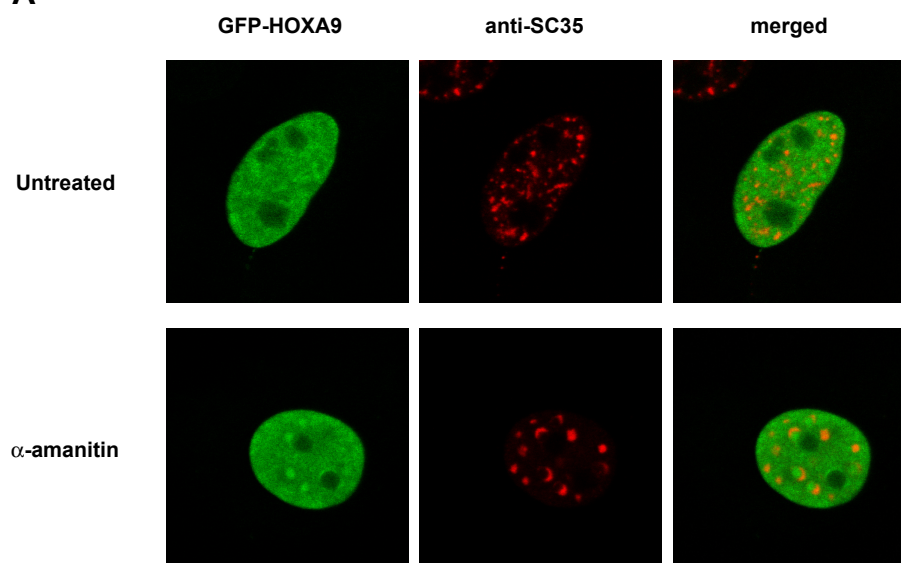

**B**

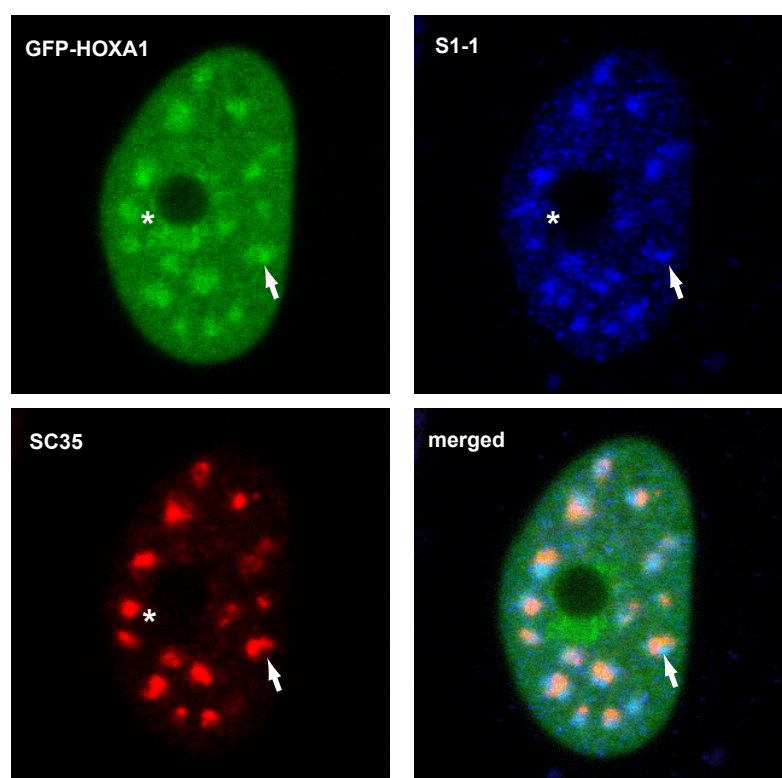

Supplement: Figure S6 — Inhibiting transcription with α-amanitin forces some His-containing transcription factors to be retained in nuclear speckles. A) HeLa cells were transfected with the expression plasmid encoding the transcription factor HOXA9. At 48 h post-transfection, the cells were treated with α-amanitin to inhibit transcription and immunostained for SC35 to assess the accumulation of both proteins in the SFC compartment (right panels). Nuclear speckles appear larger and rounder as a consequence of the treatment with the inhibitor. Note that co-localization with nuclear speckles was only observed in cells treated with α-amanitin. B) HeLa cells were transfected with pGFP-HOXA1, and double stained for S1-1 (blue) and SC35 (red). Arrows indicate some of the overlapping structures with S1-1 staining and asterisks those with SC35 staining. Images were acquired by confocal microscopy. (1.12 MB PDF) [file pgen.1000397.s006.pdf]

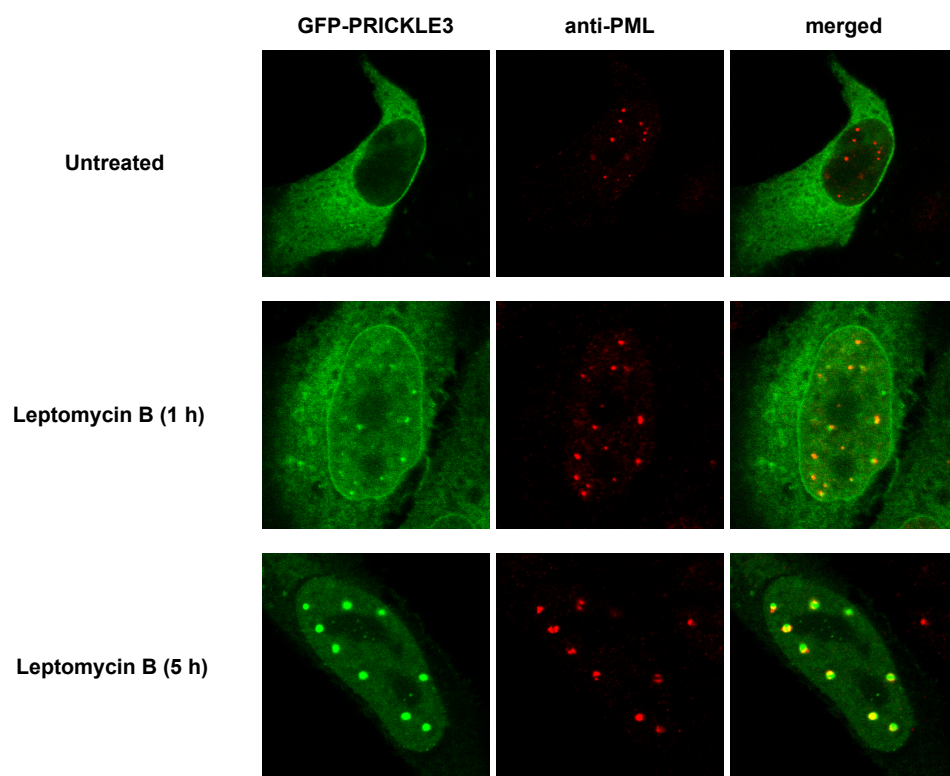

Supplement: Figure S7 — Inhibiting export with leptomycin B forces PRICKLE3 to be retained in PML bodies. HeLa cells were transfected with the expression plasmid encoding PRICKLE3. At 48 h post-transfection, cells were treated with leptomycin B for the times indicated to inhibit nuclear export, and immunostained for PML to assess accumulation in PML bodies (right panels). PML bodies appear larger and rounder as a consequence of the treatment with the inhibitor. Note that PRICKLE3 translocates to the nucleus and co-localizes with PML bodies in cells treated with leptomycin B. This behavior in response to leptomycin treatment has been also described for other proteins accumulating in the nuclear speckles compartment, such as the spliceosome component U1A or the transcription factor ZBP1. (0.87 MB PDF) [file pgen.1000397.s007.pdf]

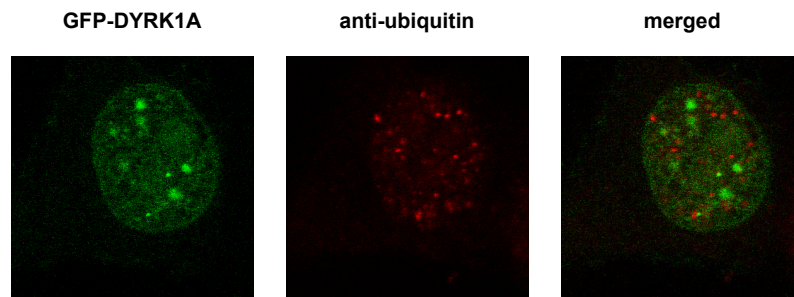

Supplement: Figure S8 — The dot-like staining of polyHis-containing proteins does not overlap with ubiquitin-enriched nuclear aggregates. HeLa cells were transfected with the expression plasmid for the fusion protein GFP-DYRK1A, and cells were immunostained for ubiquitin and then analyzed by direct fluorescence (left panel, green) and by immunofluorescence (middle panel, red). A merged image is also shown (right panel). Note that no co-localization of the DYRK1A nuclear speckles with ubiquitin was detected. (0.30 MB PDF) [file pgen.1000397.s008.pdf]

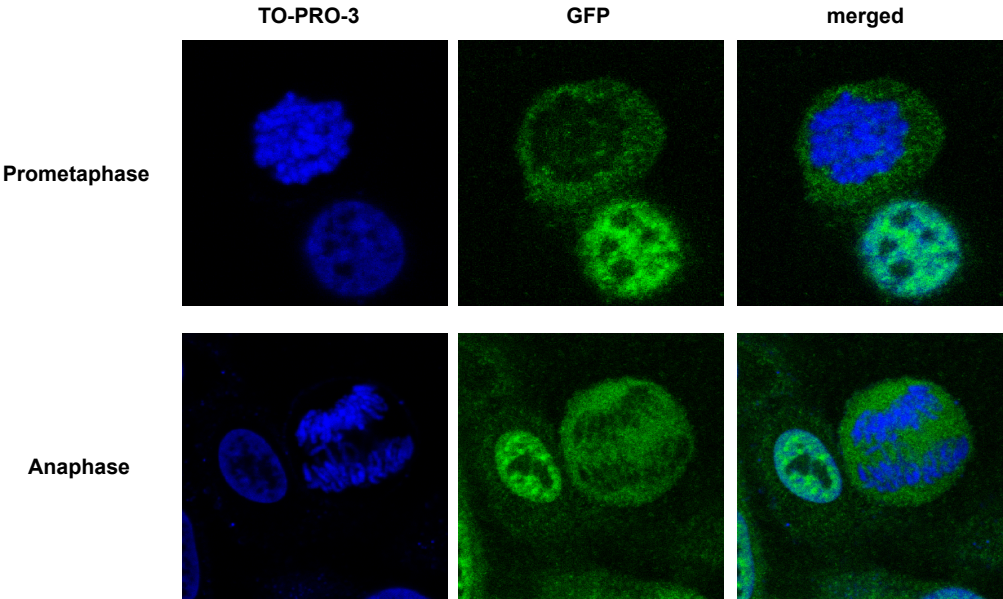

Supplement: Figure S9 — A protein with polyHis-stretches mimics the behavior of a component of endogenous nuclear speckles during the cell cycle. An U2-OS stable cell line expressing GFP fused to a fragment of the DYRK1A protein kinase (amino acids 378–616) that contains the polyHis segment was generated and the co-localization of the GFP signal with SC35 was confirmed (data not shown). Cells grown on coverslips were analyzed by direct fluorescence (central panel) and DNA was stained with TO-PRO-3 to distinguish interphase from mitotic nuclei (left panel). Note that GFP-DYRK1A(378–616) is expressed in discrete foci compatible with nuclear speckles in interphase nuclei, whereas during mitosis (prophase, upper panel; anaphase, lower panel) diffuse staining throughout the cytoplasm is observed as a consequence of nuclear speckle disassembly. This behavior mirrors that of endogenous SC35, whose speckled distribution is lost during prophase. (0.96 MB PDF) [file pgen.1000397.s009.pdf]
