# Supplementary material for: Genome-Wide Analysis of Histidine Repeats Reveals Their Role in the Localization of Human Proteins to the Nuclear Speckles Compartment
Source: PLoS Genet. 2009 Mar 6;5(3):e1000397. doi: 10.1371/journal.pgen.1000397 (PMC2644819; doi:10.1371/journal.pgen.1000397)
Supplement: Table S3 — Information on IMAGE clones and the oligonucleotides used to generate all the expression vectors. (0.06 MB PDF) [file pgen.1000397.s012.pdf]

Table S3. Information on IMAGE clones and the oligonucleotides used

Name	Accession Nº	IMAGE clone ID	Refseq ID	Forward primer	Reverse primer
CBX4	EU439707	5493993	NM_003655	5'-**AGATCT**ATGGAGCTGCCAGC-3'	5'-CGGCTACACCGTCACGTAC-3'
DLX2	BC032558	5562689	NM_004405	5'-**AGATCT**ATGACTGGAGTCTTTG-3'	5'-TCTCCCTGGGGTTAGAAAATC-3'
FAM76A	BC025768	5211010	NM_152660	5'-**AGATCT**ATGGCGGCGCTCTAC-3'	5'-GTCTGTCATGGAGAGGTTATAG-3'
FAM76B	BC028727	4824833	NM_144664	5'-**AGATCT**ATGGCGGCCTCGG-3'	5'-CACATTACATACTCCTATCTCC
FOXG1B	BC050072	5284335	NM_005249	5'-**AGATCT**TGGGTGATGCTGGAC-3'	5'-TCCCAGGGATGTTAATGTATT-3'
GSH2	EU596451	2103455	NM_133267	5'-**GGATCC**ATGTCGCGCTCCTTCTATGTCGACTCG-3'	5'-GCCGCGGCCGAGCCAGGCTGCTGGGGCTGA-3'
HAND1	BC021190	3162118	NM_004821	5'-**GGATCC**ATGAACCTCGTGGG-3'	5'-CTCGGCTCACTGGTTTAACTC-3'
HOXA1	BC032547	5537563	NM_005522	5'-**GGATCC**ATGGACAATGCAAG-3'	5'-AGCCGCCTCAGTGGGAGG-3'
HOXA9	BC006537	2987903	NM_152739	5'-**GGATCC**ATGGCCACCACTGG-3'	5'-GCCCAAATGGCATCACTCGTC-3'
MAFA	BE676631	3296459	NM_201589	5'-**AGATCT**GCCTACGAGGCTTTC-3'	5'-GGGGCCGCCCCGGCCC-3'
MAFB	BC028098	5242606	NM_005461	5'-**AGATCT**ATGGGCCGCGGAGCTG-3'	5'-CGGCCACGACTCACAGAAAG-3'
MECP2	BC011612	3956518	NM_004992	5'-**GGATCC**ATGGTAGCTGGGATG-3'	5'-GTAAAGTCAGCTAACTCTCTC-3'
MEOX2	BC017021	5209130	NM_005924	5'-**GGATCC**ATGGAACACCCGCTC-3'	5'-ATCATAAGTGCGCATGCTCTGAG-3'
MEOX2ΔHB	BC017021	5209130	NM_005924	5'**GGATCC**ATGGAACACCCGCTC-3'	5'TTTGCTCTTTGGTAAATGCTGTC-3'
NLK	BC064663	6527673	NM_016231	5'-**AGATCT**ATGGCGGCTTACAATG-3'	5'-CACCATCACTCCCACACCAG-3'
ONECUT1	EU532019	4372264	NM_004498	5'-**AGATCT**CCCACCATGACCATG-3'	5'-CTTCCTTCATGCTTTGGTAC-3'
OTX1	BC007621	3355563	NM_014562	5'-**AGATCT**GTTAGCATGATGTCT-3'	5'-ATTCCTGGGCTCACAAGACC-3'
OTX1ΔHB	BC007621	3355563	NM_014562	5'-**AGATCT**GGCCACCATCATCAC-3'	5'-ATTCCTGGGCTCACAAGACC-3'
PLK2	BC013879	3831747	NM_006622	5'-**GGATCC**ATGGAGCTTTTGCGG-3'	5'-**GGATCC**ATTCGAAAAGTCTTTCAGTTACATC-3'
POU4F2	EU439706	DKFZp434P094Q	NM_004575	(1) 5'-**AGATCT**TCCCTGAACAGCAAG-3'<br>(2) 5'-TCCCGGGCTGGCCCTG-3'	(1) 5'-TGGACACCACAGCGCCG-3'<br>(2) 5'-CTTCTAAATGCCGGCGGAATA-3'
POU4F3	BC112207	8327675	NM_002700	5'-**AGATCT**ATGATGGCCATGAACTC-3'	5'-TCAGTGGACAGCCGAATACTTC-3'
PRICKLE3	BC016856	3846372	NM_006150	5'-**GGATCC**ATGTTCGCGCGTGG-3'	5'-CTGCCTTCAAGCCACGATGC-3'
YY1	BC065366	5815774	NM_003403	5'-**AGATCT**TCAGCCATGGCCTCG-3'	5'-TTTCACTGGTTGTTTTTGGCC-3'
ZIC3	EU532020	664181	NM_003413	5'-**GGATCC**ATGACGATGCTCCTG-3'	5'-TGTGTTTGTCCTCAGACGTACC-3'
