# Supplementary material for: Genome-Wide Analysis of Histidine Repeats Reveals Their Role in the Localization of Human Proteins to the Nuclear Speckles Compartment
Source: PLoS Genet. 2009 Mar 6;5(3):e1000397. doi: 10.1371/journal.pgen.1000397 (PMC2644819; doi:10.1371/journal.pgen.1000397)
Supplement: Table S4 — Oligonucleotides used to obtain the plasmids expressing His-tracts fused to GFP. (0.05 MB PDF) [file pgen.1000397.s013.pdf]

Table S4. Oligonucleotides used to obtain the plasmids expressing His, Gln and Pro-tracts fused to GFP (restriction sites are in bold)

Name	Oligonucleotides
5xHis	5'-**AGATCT**CACCATCACCATCACTAG**GAATTC**-3'<br>5'-**GAATTC**CTAGTGATGGTGATGGTG**AGATCT**-3'
6xHis	5'-**AGATCT**CACCATCACCATCACCATTAG**GAATTC**-3'<br>5'-**GAATTC**CTAATGGTGATGGTGATGGTG**AGATCT**-3'
7xHis	5'-**AGATCT**CACCATCACCATCACCATCACTAG**GAATTC**-3'<br>5'-**GAATTC**CTAGTGATGGTGATGGTGATGGTG**AGATCT**-3'
8xHis	5'-**AGATCT**CACCATCACCATCACCATCACCATTAG**GAATTC**-3'<br>5'-**GAATTC**CTAATGGTGATGGTGATGGTGATGGTG**AGATCT**-3'
9xHis	5'-**AGATCT**CACCATCACCATCACCATCACCATCACTAG**GAATTC**-3'<br>5'-**GAATTC**CTAGTGATGGTGATGGTGATGGTGATGGTG**AGATCT**-3'
9xGln	5'-**AGATCT**CAACAGCAACAGCAACAGCAACAGCAATAG**GAATTC**-3'<br>5'-**GAATTC**CTATTGCTGTTGCTGTTGCTGTTGCTGTTG**AGATCT**-3'
9xPro	5'-**AGATCT**CCACCGCCACCGCCACCGCCACCGCCATAG**GAATTC**-3'<br>5'-**GAATTC**CTATGGCGGTGGCGGTGGCGGTGGCGGTGG**AGATCT**-3'
