# Supplementary material for: Genome-Wide Analysis of Histidine Repeats Reveals Their Role in the Localization of Human Proteins to the Nuclear Speckles Compartment
Source: PLoS Genet. 2009 Mar 6;5(3):e1000397. doi: 10.1371/journal.pgen.1000397 (PMC2644819; doi:10.1371/journal.pgen.1000397)
Supplement: Text S1 — Supporting Materials and Methods. (0.07 MB PDF) [file pgen.1000397.s014.pdf]

SUPPORTING MATERIALS AND METHODS

The identification number of the IMAGE clones used to generate the expression plasmids for the GFP fusion proteins is listed in Table S3, in which the sequence of the forward and reverse primers used to obtain the corresponding PCR product is also included. The Accession Nº corresponds to the sequence present in the databank of the IMAGE clone. Some of the IMAGE clones were completely sequenced in this study (CBX4, GSH2, ONECUT1, POU4F2 and ZIC3) and their corresponding DNA sequences were submitted to GenBank under the Accession Nº indicated. Taq polymerase from the Expand High Fidelity PCR System (Roche) was used for amplification, except for the POU4F2 (fragment 1), FOXG1B, MAFB and YY1 PCR products where the GC-RICH PCR System (Roche) was used. The HAND1 open reading frame was amplified with the PCRx Enhancer System (Invitrogen). In the case of GSH2, the PCR reaction was carried out with the Expand Long Template PCR System (Roche). The DNA fragments were cloned into pGEM-TEasy, sequenced and later subcloned into the appropriate restriction sites of pEGFP-C1 (Clontech). The cDNA of POU4F2 was amplified separately in two PCR products (fragment 1: *Bgl*II/*Apa*I and fragment 2: *Apa*I/*EcoR*I) that were then ligated together into the *Bgl*II and *EcoR*I sites of pEGFP-C1. The expression plasmid for GFP-POU4F1 was obtained by ligating an *EcoRI/BamHI* fragment of pTS-Brn3a into the appropriate restriction sites of pEGFP-C1.
